# Precision treatment of beta-cell monogenic diabetes: a systematic review

**DOI:** 10.1038/s43856-024-00556-1

**Published:** 2024-07-18

**Authors:** Rochelle N. Naylor, Kashyap A. Patel, Jarno L. T. Kettunen, Jonna M. E. Männistö, Julie Støy, Jacques Beltrand, Michel Polak, ADA/EASD PMDI, Deirdre K. Tobias, Jordi Merino, Abrar Ahmad, Catherine Aiken, Jamie L. Benham, Dhanasekaran Bodhini, Amy L. Clark, Kevin Colclough, Rosa Corcoy, Sara J. Cromer, Daisy Duan, Jamie L. Felton, Ellen C. Francis, Pieter Gillard, Véronique Gingras, Romy Gaillard, Eram Haider, Alice Hughes, Jennifer M. Ikle, Laura M. Jacobsen, Anna R. Kahkoska, Raymond J. Kreienkamp, Lee-Ling Lim, Robert Massey, Niamh-Maire Mclennan, Rachel G. Miller, Mario Luca Morieri, Jasper Most, Bige Ozkan, Kashyap Amratlal Patel, Scott J. Pilla, Katsiaryna Prystupa, Sridharan Raghavan, Mary R. Rooney, Martin Schön, Zhila Semnani-Azad, Magdalena Sevilla-Gonzalez, Pernille Svalastoga, Wubet Worku Takele, Claudia Ha-ting Tam, Anne Cathrine B. Thuesen, Mustafa Tosur, Amelia S. Wallace, Caroline C. Wang, Jessie J. Wong, Jennifer M. Yamamoto, Katherine Young, Chloé Amouyal, Mette K. Andersen, Maxine P. Bonham, Mingling Chen, Feifei Cheng, Tinashe Chikowore, Sian C. Chivers, Christoffer Clemmensen, Dana Dabelea, Adem Y. Dawed, Aaron J. Deutsch, Laura T. Dickens, Linda A. DiMeglio, Monika Dudenhöffer-Pfeifer, Carmella Evans-Molina, María Mercè Fernández-Balsells, Hugo Fitipaldi, Stephanie L. Fitzpatrick, Stephen E. Gitelman, Mark O. Goodarzi, Jessica A. Grieger, Marta Guasch-Ferré, Nahal Habibi, Torben Hansen, Chuiguo Huang, Arianna Harris-Kawano, Heba M. Ismail, Benjamin Hoag, Randi K. Johnson, Angus G. Jones, Robert W. Koivula, Aaron Leong, Gloria K. W. Leung, Ingrid M. Libman, Kai Liu, S. Alice Long, William L. Lowe, Robert W. Morton, Ayesha A. Motala, Suna Onengut-Gumuscu, James S. Pankow, Maleesa Pathirana, Sofia Pazmino, Dianna Perez, John R. Petrie, Camille E. Powe, Alejandra Quinteros, Rashmi Jain, Debashree Ray, Mathias Ried-Larsen, Zeb Saeed, Vanessa Santhakumar, Sarah Kanbour, Sudipa Sarkar, Gabriela S. F. Monaco, Denise M. Scholtens, Elizabeth Selvin, Wayne Huey-Herng Sheu, Cate Speake, Maggie A. Stanislawski, Nele Steenackers, Andrea K. Steck, Norbert Stefan, Julie Støy, Rachael Taylor, Sok Cin Tye, Gebresilasea Gendisha Ukke, Marzhan Urazbayeva, Bart Van der Schueren, Camille Vatier, John M. Wentworth, Wesley Hannah, Sara L. White, Gechang Yu, Yingchai Zhang, Shao J. Zhou, Jacques Beltrand, Michel Polak, Ingvild Aukrust, Elisa de Franco, Sarah E. Flanagan, Kristin A. Maloney, Andrew McGovern, Janne Molnes, Mariam Nakabuye, Pål Rasmus Njølstad, Hugo Pomares-Millan, Michele Provenzano, Cécile Saint-Martin, Cuilin Zhang, Yeyi Zhu, Sungyoung Auh, Russell de Souza, Andrea J. Fawcett, Chandra Gruber, Eskedar Getie Mekonnen, Emily Mixter, Diana Sherifali, Robert H. Eckel, John J. Nolan, Louis H. Philipson, Rebecca J. Brown, Liana K. Billings, Kristen Boyle, Tina Costacou, John M. Dennis, Jose C. Florez, Anna L. Gloyn, Maria F. Gomez, Peter A. Gottlieb, Siri Atma W. Greeley, Kurt Griffin, Andrew T. Hattersley, Irl B. Hirsch, Marie-France Hivert, Korey K. Hood, Jami L. Josefson, Soo Heon Kwak, Lori M. Laffel, Siew S. Lim, Ruth J. F. Loos, Ronald C. W. Ma, Chantal Mathieu, Nestoras Mathioudakis, James B. Meigs, Shivani Misra, Viswanathan Mohan, Rinki Murphy, Richard Oram, Katharine R. Owen, Susan E. Ozanne, Ewan R. Pearson, Wei Perng, Toni I. Pollin, Rodica Pop-Busui, Richard E. Pratley, Leanne M. Redman, Maria J. Redondo, Rebecca M. Reynolds, Robert K. Semple, Jennifer L. Sherr, Emily K. Sims, Arianne Sweeting, Tiinamaija Tuomi, Miriam S. Udler, Kimberly K. Vesco, Tina Vilsbøll, Robert Wagner, Stephen S. Rich, Paul W. Franks, Tina Vilsbøll, Siri A. W. Greeley, Andrew T. Hattersley, Tiinamaija Tuomi

**Affiliations:** 1 Departments of Pediatrics and Medicine, University of Chicago, Chicago, IL USA; 2 University of Exeter Medical School, Department of Clinical and Biomedical Sciences, Exeter, Devon UK; 3 Helsinki University Hospital, Abdominal Centre/Endocrinology, Helsinki, Finland; 4 Folkhalsan Research Center, Helsinki, Finland; 5 Institute for Molecular Medicine Finland FIMM, University of Helsinki, Helsinki, Finland; 6 Departments of Pediatrics and Clinical Genetics, Kuopio University Hospital, Kuopio, Finland; 7 Department of Medicine, University of Eastern Finland, Kuopio, Finland; 8 Steno diabetes center Aarhus, Aarhus University Hospital, Aarhus, Denmark; 9 APHP Centre Hôpital Necker Enfants Malades Université Paris Cité, Paris, France; 10 Inserm U1016 Institut Cochin, Paris, France; 11 Department of Pediatric Endocrinology, Gynecology and Diabetology, Hôpital Universitaire Necker Enfants Malades, Paris, France; 12 Université Paris Cité, Paris, France; 13 Department of Clinical Medicine, University of Copenhagen, København, Denmark; 14 Lund University Diabetes Center, Malmo, Sweden; 15 Division of Preventative Medicine, Department of Medicine, Brigham and Women’s Hospital and Harvard Medical School, Boston, MA USA; 16 Department of Nutrition, Harvard T.H. Chan School of Public Health, Boston, MA USA; 17 Novo Nordisk Foundation Center for Basic Metabolic Research, Faculty of Health and Medical Sciences, University of Copenhagen, Copenhagen, Denmark; 18 Diabetes Unit, Endocrine Division, Massachusetts General Hospital, Boston, MA USA; 19 Center for Genomic Medicine, Massachusetts General Hospital, Boston, MA USA; 20 Department of Clinical Sciences, Lund University Diabetes Centre, Lund University, Malmö, Sweden; 21 Department of Obstetrics and Gynaecology, the Rosie Hospital, Cambridge, UK; 22 NIHR Cambridge Biomedical Research Centre, University of Cambridge, Cambridge, UK; 23 Departments of Medicine and Community Health Sciences, Cumming School of Medicine, University of Calgary, Calgary, AB Canada; 24 Department of Molecular Genetics, Madras Diabetes Research Foundation, Chennai, India; 25 Division of Pediatric Endocrinology, Department of Pediatrics, Saint Louis University School of Medicine, SSM Health Cardinal Glennon Children’s Hospital, St. Louis, MO USA; 26 Department of Clinical and Biomedical Sciences, University of Exeter Medical School, Exeter, Devon, UK; 27 CIBER-BBN, ISCIII, Madrid, Spain; 28 Institut d’Investigació Biomèdica Sant Pau (IIB SANT PAU), Barcelona, Spain; 29 Departament de Medicina, Universitat Autònoma de Barcelona, Bellaterra, Spain; 30 Programs in Metabolism and Medical & Population Genetics, Broad Institute, Cambridge, MA USA; 31 Department of Medicine, Harvard Medical School, Boston, MA USA; 32 Division of Endocrinology, Diabetes and Metabolism, Johns Hopkins University School of Medicine, Baltimore, MD USA; 33 Department of Pediatrics, Indiana University School of Medicine, Indianapolis, IN USA; 34 Herman B Wells Center for Pediatric Research, Indiana University School of Medicine, Indianapolis, IN USA; 35 Center for Diabetes and Metabolic Diseases, Indiana University School of Medicine, Indianapolis, IN USA; 36 Department of Biostatistics and Epidemiology, Rutgers School of Public Health, Piscataway, NJ USA; 37 University Hospital Leuven, Leuven, Belgium; 38 Department of Nutrition, Université de Montréal, Montreal, QC Canada; 39 Research Center, Sainte-Justine University Hospital Center, Montreal, QC Canada; 40 Department of Pediatrics, Erasmus Medical Center, Rotterdam, The Netherlands; 41 Division of Population Health & Genomics, School of Medicine, University of Dundee, Dundee, UK; 42 Department of Pediatrics, Stanford School of Medicine, Stanford University, Stanford, CA USA; 43 Stanford Diabetes Research Center, Stanford School of Medicine, Stanford University, Stanford, CA USA; 44 University of Florida, Gainesville, FL USA; 45 Department of Nutrition, University of North Carolina at Chapel Hill, Chapel Hill, NC USA; 46 Department of Pediatrics, Division of Endocrinology, Boston Children’s Hospital, Boston, MA USA; 47 Department of Medicine, Faculty of Medicine, University of Malaya, Kuala Lumpur, Malaysia; 48 Asia Diabetes Foundation, Hong Kong SAR, China; 49 Department of Medicine & Therapeutics, Chinese University of Hong Kong, Hong Kong SAR, China; 50 Centre for Cardiovascular Science, Queen’s Medical Research Institute, University of Edinburgh, Edinburgh, UK; 51 Department of Epidemiology, University of Pittsburgh, Pittsburgh, PA USA; 52 Metabolic Disease Unit, University Hospital of Padova, Padova, Italy; 53 Department of Medicine, University of Padova, Padova, Italy; 54 Department of Orthopedics, Zuyderland Medical Center, Sittard-Geleen, The Netherlands; 55 Welch Center for Prevention, Epidemiology, and Clinical Research, Johns Hopkins Bloomberg School of Public Health, Baltimore, MD USA; 56 Ciccarone Center for the Prevention of Cardiovascular Disease, Johns Hopkins School of Medicine, Baltimore, MD USA; 57 Department of Medicine, Johns Hopkins University, Baltimore, MD USA; 58 Department of Health Policy and Management, Johns Hopkins University Bloomberg School of Public Health, Baltimore, MD USA; 59 Institute for Clinical Diabetology, German Diabetes Center, Leibniz Center for Diabetes Research at Heinrich Heine University Düsseldorf, Düsseldorf, Germany; 60 German Center for Diabetes Research (DZD), Ingolstädter Landstraße 1, 85764 Neuherberg, Germany; 61 Section of Academic Primary Care, US Department of Veterans Affairs Eastern Colorado Health Care System, Aurora, CO USA; 62 Department of Medicine, University of Colorado School of Medicine, Aurora, CO USA; 63 Department of Epidemiology, Johns Hopkins Bloomberg School of Public Health, Baltimore, MD USA; 64 Institute of Experimental Endocrinology, Biomedical Research Center, Slovak Academy of Sciences, Bratislava, Slovakia; 65 Clinical and Translational Epidemiology Unit, Massachusetts General Hospital, Boston, MA USA; 66 Mohn Center for Diabetes Precision Medicine, Department of Clinical Science, University of Bergen, Bergen, Norway; 67 Children and Youth Clinic, Haukeland University Hospital, Bergen, Norway; 68 Eastern Health Clinical School, Monash University, Melbourne, VIC Australia; 69 Laboratory for Molecular Epidemiology in Diabetes, Li Ka Shing Institute of Health Sciences, The Chinese University of Hong Kong, Hong Kong, China; 70 Hong Kong Institute of Diabetes and Obesity, The Chinese University of Hong Kong, Hong Kong, China; 71 Department of Pediatrics, Baylor College of Medicine, Houston, TX USA; 72 Division of Pediatric Diabetes and Endocrinology, Texas Children’s Hospital, Houston, TX USA; 73 Children’s Nutrition Research Center, USDA/ARS, Houston, TX USA; 74 Stanford University School of Medicine, Stanford, CA USA; 75 Internal Medicine, University of Manitoba, Winnipeg, MB Canada; 76 Department of Diabetology, APHP, Paris, France; 77 Sorbonne Université, INSERM, NutriOmic team, Paris, France; 78 Department of Nutrition, Dietetics and Food, Monash University, Melbourne, VIC Australia; 79 Monash Centre for Health Research and Implementation, Monash University, Clayton, VIC Australia; 80 Health Management Center, The Second Affiliated Hospital of Chongqing Medical University, Chongqing Medical University, Chongqing, China; 81 MRC/Wits Developmental Pathways for Health Research Unit, Department of Paediatrics, Faculty of Health Sciences, University of the Witwatersrand, Johannesburg, South Africa; 82 Channing Division of Network Medicine, Brigham and Women’s Hospital, Boston, MA USA; 83 Sydney Brenner Institute for Molecular Bioscience, Faculty of Health Sciences, University of the Witwatersrand, Johannesburg, South Africa; 84 Department of Women and Children’s health, King’s College London, London, UK; 85 Lifecourse Epidemiology of Adiposity and Diabetes (LEAD) Center, University of Colorado Anschutz Medical Campus, Aurora, CO USA; 86 Section of Adult and Pediatric Endocrinology, Diabetes and Metabolism, Kovler Diabetes Center, University of Chicago, Chicago, IL USA; 87 Department of Pediatrics, Riley Hospital for Children, Indiana University School of Medicine, Indianapolis, IN USA; 88 Richard L. Roudebush VAMC, Indianapolis, IN USA; 89 Biomedical Research Institute Girona, IdIBGi, Girona, Spain; 90 Diabetes, Endocrinology and Nutrition Unit Girona, University Hospital Dr Josep Trueta, Girona, Spain; 91 Institute of Health System Science, Feinstein Institutes for Medical Research, Northwell Health, Manhasset, NY USA; 92 University of California at San Francisco, Department of Pediatrics, Diabetes Center, San Francisco, CA USA; 93 Division of Endocrinology, Diabetes and Metabolism, Cedars-Sinai Medical Center, Los Angeles, CA USA; 94 Department of Medicine, Cedars-Sinai Medical Center, Los Angeles, CA USA; 95 Adelaide Medical School, Faculty of Health and Medical Sciences, The University of Adelaide, Adelaide, SA Australia; 96 Robinson Research Institute, The University of Adelaide, Adelaide, SA Australia; 97 Department of Public Health and Novo Nordisk Foundation Center for Basic Metabolic Research, Faculty of Health and Medical Sciences, University of Copenhagen, Copenhagen, Denmark; 98 Division of Endocrinology and Diabetes, Department of Pediatrics, Sanford Children’s Hospital, Sioux Falls, SD USA; 99 University of South Dakota School of Medicine, E Clark St, Vermillion, SD USA; 100 Department of Biomedical Informatics, University of Colorado Anschutz Medical Campus, Aurora, CO USA; 101 Department of Epidemiology, Colorado School of Public Health, Aurora, CO USA; 102 Royal Devon University Healthcare NHS Foundation Trust, Exeter, UK; 103 Oxford Centre for Diabetes, Endocrinology and Metabolism, University of Oxford, Oxford, UK; 104 Division of General Internal Medicine, Massachusetts General Hospital, Boston, MA USA; 105 UPMC Children’s Hospital of Pittsburgh, Pittsburgh, PA USA; 106 Center for Translational Immunology, Benaroya Research Institute, Seattle, WA USA; 107 Department of Medicine, Northwestern University Feinberg School of Medicine, Chicago, IL USA; 108 Department of Pathology & Molecular Medicine, McMaster University, Hamilton, ON Canada; 109 Population Health Research Institute, Hamilton, ON Canada; 110 Department of Translational Medicine, Medical Science, Novo Nordisk Foundation, Hellerup, Denmark; 111 Department of Diabetes and Endocrinology, Nelson R. Mandela School of Medicine, University of KwaZulu-Natal, Durban, South Africa; 112 Center for Public Health Genomics, Department of Public Health Sciences, University of Virginia, Charlottesville, VA USA; 113 Division of Epidemiology and Community Health, School of Public Health, University of Minnesota, Minnesota, MN USA; 114 Department of Chronic Diseases and Metabolism, Clinical and Experimental Endocrinology, KU Leuven, Leuven Belgium; 115 School of Health and Wellbeing, College of Medical, Veterinary and Life Sciences, University of Glasgow, Glasgow, UK; 116 Department of Obstetrics, Gynecology, and Reproductive Biology, Massachusetts General Hospital and Harvard Medical School, Boston, MA USA; 117 Sanford Children’s Specialty Clinic, Sioux Falls, SD USA; 118 Department of Pediatrics, Sanford School of Medicine, University of South Dakota, Sioux Falls, SD USA; 119 Department of Biostatistics, Johns Hopkins Bloomberg School of Public Health, Baltimore, MD USA; 120 Centre for Physical Activity Research, Rigshospitalet, Copenhagen Denmark; 121 Institute for Sports and Clinical Biomechanics, University of Southern Denmark, Odense, Denmark; 122 Department of Medicine, Division of Endocrinology, Diabetes and Metabolism, Indiana University School of Medicine, Indianapolis, IN USA; 123 AMAN Hospital, Doha, Qatar; 124 Department of Preventive Medicine, Division of Biostatistics, Northwestern University Feinberg School of Medicine, Chicago, IL USA; 125 Institute of Molecular and Genomic Medicine, National Health Research Institutes, Taipei City, Taiwan; 126 Divsion of Endocrinology and Metabolism, Taichung Veterans General Hospital, Taichung, Taiwan; 127 Division of Endocrinology and Metabolism, Taipei Veterans General Hospital, Taipei, Taiwan; 128 Center for Interventional Immunology, Benaroya Research Institute, Seattle, WA USA; 129 Barbara Davis Center for Diabetes, University of Colorado Anschutz Medical Campus, Aurora, CO USA; 130 University Hospital of Tübingen, Tübingen, Germany; 131 Institute of Diabetes Research and Metabolic Diseases (IDM), Helmholtz Center Munich, Neuherberg, Germany; 132 Steno Diabetes Center Aarhus, Aarhus University Hospital, Aarhus, Denmark; 133 University of Newcastle, Newcastle upon Tyne, UK; 134 Sections on Genetics and Epidemiology, Joslin Diabetes Center, Harvard Medical School, Boston, MA USA; 135 Department of Clinical Pharmacy and Pharmacology, University Medical Center Groningen, Groningen, The Netherlands; 136 Gastroenterology, Baylor College of Medicine, Houston, TX USA; 137 Department of Endocrinology, University Hospitals Leuven, Leuven, Belgium; 138 Sorbonne University, Inserm U938, Saint-Antoine Research Centre, Institute of Cardiometabolism and Nutrition, Paris, France; 139 Department of Endocrinology, Diabetology and Reproductive Endocrinology, Assistance Publique-Hôpitaux de Paris, Saint-Antoine University Hospital, National Reference Center for Rare Diseases of Insulin Secretion and Insulin Sensitivity (PRISIS), Paris, France; 140 Royal Melbourne Hospital Department of Diabetes and Endocrinology, Parkville, VIC Australia; 141 Walter and Eliza Hall Institute, Parkville, VIC Australia; 142 University of Melbourne Department of Medicine, Parkville, VIC Australia; 143 Deakin University, Melbourne, VIC Australia; 144 Department of Epidemiology, Madras Diabetes Research Foundation, Chennai, India; 145 Department of Diabetes and Endocrinology, Guy’s and St Thomas’ Hospitals NHS Foundation Trust, London, UK; 146 School of Agriculture, Food, and Wine, University of Adelaide, Adelaide, SA Australia; 147 Institut Cochin, Inserm U 10116 Paris, France; 148 Pediatric Endocrinology and Diabetes, Hopital Necker Enfants Malades, APHP Centre, Université de Paris, Paris, France; 149 Department of Medical Genetics, Haukeland University Hospital, Bergen, Norway; 150 Department of Medicine, University of Maryland School of Medicine, Baltimore, MD USA; 151 Department of Epidemiology, Geisel School of Medicine at Dartmouth, Hanover, NH USA; 152 Nephrology, Dialysis and Renal Transplant Unit, IRCCS—Azienda Ospedaliero-Universitaria di Bologna, Alma Mater Studiorum University of Bologna, Bologna, Italy; 153 Department of Medical Genetics, AP-HP Pitié-Salpêtrière Hospital, Sorbonne University, Paris, France; 154 Global Center for Asian Women’s Health, Yong Loo Lin School of Medicine, National University of Singapore, Singapore, Singapore; 155 Department of Obstetrics and Gynecology, Yong Loo Lin School of Medicine, National University of Singapore, Singapore, Singapore; 156 Kaiser Permanente Northern California Division of Research, Oakland, Oakland, CA USA; 157 Department of Epidemiology and Biostatistics, University of California San Francisco, San Francisco, CA USA; 158 National Institute of Diabetes and Digestive and Kidney Diseases, National Institutes of Health, Bethesda, MD USA; 159 Department of Health Research Methods, Evidence, and Impact, Faculty of Health Sciences, McMaster University, Hamilton, ON Canada; 160 Ann & Robert H. Lurie Children’s Hospital of Chicago, Department of Pediatrics, Northwestern University Feinberg School of Medicine, Chicago, IL USA; 161 Department of Clinical and Organizational Development, Chicago, IL USA; 162 American Diabetes Association, Arlington, VA USA; 163 College of Medicine and Health Sciences, University of Gondar, Gondar, Ethiopia; 164 Global Health Institute, Faculty of Medicine and Health Sciences, University of Antwerp, 2160 Antwerp, Belgium; 165 Department of Medicine and Kovler Diabetes Center, University of Chicago, Chicago, IL USA; 166 School of Nursing, Faculty of Health Sciences, McMaster University, Hamilton, ON Canada; 167 Division of Endocrinology, Metabolism, Diabetes, University of Colorado, Boulder, CO USA; 168 Department of Clinical Medicine, School of Medicine, Trinity College Dublin, Dublin, Ireland; 169 Department of Endocrinology, Wexford General Hospital, Wexford, Ireland; 170 Division of Endocrinology, NorthShore University HealthSystem, Skokie, IL USA; 171 Department of Medicine, Prtizker School of Medicine, University of Chicago, Chicago, IL USA; 172 Department of Genetics, Stanford School of Medicine, Stanford University, Stanford, CA USA; 173 Faculty of Health, Aarhus University, Aarhus, Denmark; 174 Departments of Pediatrics and Medicine and Kovler Diabetes Center, University of Chicago, Chicago, IL USA; 175 Sanford Research, Sioux Falls, SD USA; 176 University of Washington School of Medicine, Seattle, WA USA; 177 Department of Population Medicine, Harvard Medical School, Harvard Pilgrim Health Care Institute, Boston, MA USA; 178 Department of Medicine, Universite de Sherbrooke, Sherbrooke, QC Canada; 179 Department of Internal Medicine, Seoul National University College of Medicine, Seoul National University Hospital, Seoul, Republic of Korea; 180 Joslin Diabetes Center, Harvard Medical School, Boston, MA USA; 181 Charles Bronfman Institute for Personalized Medicine, Icahn School of Medicine at Mount Sinai, New York, NY USA; 182 Broad Institute, Cambridge, MA USA; 183 Division of Metabolism, Digestion and Reproduction, Imperial College London, London, UK; 184 Department of Diabetes & Endocrinology, Imperial College Healthcare NHS Trust, London, UK; 185 Department of Diabetology, Madras Diabetes Research Foundation & Dr. Mohan’s Diabetes Specialities Centre, Chennai, India; 186 Department of Medicine, Faculty of Medicine and Health Sciences, University of Auckland, Auckland, New Zealand; 187 Auckland Diabetes Centre, Te Whatu Ora Health New Zealand, Auckland, New Zealand; 188 Medical Bariatric Service, Te Whatu Ora Counties, Health New Zealand, Auckland, New Zealand; 189 Oxford NIHR Biomedical Research Centre, University of Oxford, Oxford, UK; 190 University of Cambridge, Metabolic Research Laboratories and MRC Metabolic Diseases Unit, Wellcome-MRC Institute of Metabolic Science, Cambridge, UK; 191 Department of Epidemiology & Public Health, University of Maryland School of Medicine, Baltimore, MD USA; 192 Department of Internal Medicine, Division of Metabolism, Endocrinology and Diabetes, University of Michigan, Ann Arbor, MI USA; 193 AdventHealth Translational Research Institute, Orlando, FL USA; 194 Pennington Biomedical Research Center, Baton Rouge, LA USA; 195 MRC Human Genetics Unit, Institute of Genetics and Cancer, University of Edinburgh, Edinburgh, UK; 196 Yale School of Medicine, New Haven, CT USA; 197 Faculty of Medicine and Health, University of Sydney, Sydney, NSW Australia; 198 Department of Endocrinology, Royal Prince Alfred Hospital, Sydney, NSW Australia; 199 Kaiser Permanente Northwest, Kaiser Permanente Center for Health Research, Portland, OR USA; 200 Clinial Research, Steno Diabetes Center Copenhagen, Herlev, Denmark; 201 Department of Clinical Medicine, Faculty of Health and Medical Sciences, University of Copenhagen, Copenhagen, Denmark; 202 Department of Endocrinology and Diabetology, University Hospital Düsseldorf, Heinrich Heine University Düsseldorf, Düsseldorf, Germany

**Keywords:** Endocrinology, Genetics

## Abstract

**Background:**

Beta-cell monogenic forms of diabetes have strong support for precision medicine. We systematically analyzed evidence for precision treatments for GCK-related hyperglycemia, HNF1A-, HNF4A- and HNF1B-diabetes, and mitochondrial diabetes (MD) due to m.3243 A > G variant, 6q24-transient neonatal diabetes mellitus (TND) and SLC19A2-diabetes.

**Methods:**

The search of PubMed, MEDLINE, and Embase for individual and group level data for glycemic outcomes using inclusion (English, original articles written after 1992) and exclusion (VUS, multiple diabetes types, absent/aggregated treatment effect measures) criteria. The risk of bias was assessed using NHLBI study-quality assessment tools. Data extracted from Covidence were summarized and presented as descriptive statistics in tables and text.

**Results:**

There are 146 studies included, with only six being experimental studies. For GCK-related hyperglycemia, the six studies (35 individuals) assessing therapy discontinuation show no HbA1c deterioration. A randomized trial (18 individuals per group) shows that sulfonylureas (SU) were more effective in HNF1A-diabetes than in type 2 diabetes. Cohort and case studies support SU’s effectiveness in lowering HbA1c. Two cross-over trials (each with 15–16 individuals) suggest glinides and GLP-1 receptor agonists might be used in place of SU. Evidence for HNF4A-diabetes is limited. Most reported patients with HNF1B-diabetes (*N* = 293) and MD (*N* = 233) are on insulin without treatment studies. Limited data support oral agents after relapse in 6q24-TND and for thiamine improving glycemic control and reducing/eliminating insulin requirement in SLC19A2-diabetes.

**Conclusion:**

There is limited evidence, and with moderate or serious risk of bias, to guide monogenic diabetes treatment. Further evidence is needed to examine the optimum treatment in monogenic subtypes.

## Plain language summary

Monogenic diabetes is a type of diabetes caused by changes in genes that affect how the body makes or responds to insulin. Precision medicine (where knowledge of the gene change directs the selection of treatment) is available for some forms of monogenic diabetes. This study evaluated the published literature for several forms of monogenic diabetes to assess the level of evidence supporting specific precision treatments. Among the 146 small studies that we reviewed, only six compared different treatments. However, we found evidence supporting oral medications for some types of monogenic diabetes, and evidence that treatment is not needed for one particular type. Based on our results, we provide treatment recommendations for certain forms of monogenic diabetes and identify future directions for research to help us optimize precision medicine in monogenic diabetes.

## Abstract (web summary)

Naylor et al. systematically review the efficacy of treatments for beta-cell monogenic diabetes. Limited evidence from the mostly non-randomized, small studies supports no treatment in glucokinase-related hyperglycemia and sulfonylureas for HNF1A-diabetes; further evidence is needed on the optimum treatments in these and other monogenic subtypes.

## Introduction

In monogenic forms of diabetes, the underlying genetic cause has implications for the disease mechanism, treatment, and prognosis. Defining the underlying genetic etiology also defines the pathophysiology resulting in hyperglycemia; this greatly increases the chances of finding an optimal specific therapy or therapy for glucose lowering. Pathogenic variations in single genes can either result in reduced insulin secretion as seen in the beta-cell subtypes or reduced insulin action in the insulin-resistant subtypes. This systematic review relates to the treatment of beta-cell subtypes with the treatment of insulin-resistant subtypes being reviewed elsewhere^1^.

Beta-cell monogenic forms of diabetes have been the area of diabetes care where there is the strongest support for a precision medicine approach for treating hyperglycemia^2,3^. The supporting evidence usually came from initial case reports leading to follow-up with case series and in some cases experimental studies/trials. The evidence base is considered strong in the commonest subtypes of monogenic diabetes such as glucokinase (GCK)-related hyperglycemia, Hepatic Nuclear Factor 1 alpha (HNF1A)-diabetes, and ATP sensitive potassium channel (KATP)-neonatal diabetes (ND, related to pathogenic variants in *KCNJ11* and *ABCC8*). Such evidence is yet to be shown for the more rare subtypes of monogenic diabetes. The varying levels of evidence combined with an expert opinion have led to recommendations for optimum treatment in international guidelines such as the International Society for Pediatric and Adolescent Diabetes (ISPAD)^4^, with the strongest support for sulfonylureas (SU) as first-line therapy for HNF1A-diabetes and Hepatic Nuclear Factor 4 alpha (HNF4A)-diabetes, no pharmacologic therapy for GCK-related hyperglycemia, insulin for Hepatic Nuclear Factor 1 beta (HNF1B)-diabetes and mitochondrial diabetes (MD) and high-dose SU for KATP-ND. A key point is that these clinical guidelines were developed mostly without a systematic review of all the available evidence.

The systematic review will allow a comprehensive assessment of the strength of the evidence for the specific recommendations for precision medicine approaches. To our knowledge there is only one area of beta-cell monogenic diabetes where robust systematic reviews have been used – this is in the glycemic treatment of KATP-ND with high-dose SU^5^ and the partial response of neurological features in KATP-ND to high-dose SU therapy^6^. We therefore decided to systematically review the evidence of a precision medicine approach with an optimal glucose-lowering therapy for all the common subtypes of beta-cell monogenic diabetes except for the KATP-ND (Table 1).

**Table 1:** Subtypes of beta-call monogenic diabetes included in this systematic review

Monogenic diabetes subtype	Typical clinical features	Typical treatment approaches
GCK-related hyperglycemia	● Mild, stable hyperglycemia present from birth ● Often incidentally diagnosed during routine clinical exams or during gestational diabetes screening in pregnancy ● Fasting blood glucose typically ranges between 5.5–8.0 mmol/L and HbA1c between 5.6–7.8%	No pharmacologic treatment
HNF1A-diabetes	● Progressive insulin secretory defect with onset of diabetes typically in the second and third decades of life ● Reported to have a lowered renal threshold for glycosuria, which may be an early sign	Sensitive to sulfonylureas
HNF4A-diabetes	● Progressive insulin secretory defect with onset typically in the second and third decades of life ● May have fetal macrosomia and hyperinsulinemic hypoglycemia in the neonatal and early-childhood period	Sensitive to sulfonylureas
HNF1B-diabetes	● Syndromic form of diabetes with onset in the second and third decades of life, but typically later than in HNF1A- diabetes and HNF4A-diabetes ● Often with renal cysts or other developmental renal diseases (single kidney, horseshoe kidney) ● Other features can include hypoplasia of the pancreas, pancreatic exocrine deficiency, genital tract and biliary abnormalities, hypomagnesemia, and neurodevelopmental disorders (with whole-gene deletions)	Use of OHA reported, most cases insulin-treated
m.3243A Mitochondrial diabetes	● Maternally-inherited syndromic form of diabetes, often with sensorineural deafness ● Diabetes typically occurs in the 30 s but onset ranges from 11–68 years ● Other features can include cardiomyopathy, myopathy, epilepsy, lactatemia, macular dystrophy, renal disease (e.g., focal segmental glomerular sclerosis)	Use of OHA reported, most cases insulin-treated
6q24 Transient neonatal diabetes	● Neonatal onset of diabetes usually within the first week of life, typically associated with severe intrauterine growth restriction and small for gestation age at birth ● May have macroglossia and umbilical hernia ● Diabetes resolves by 18 months of age (average duration is 3 months) ● Diabetes may relapse in adolescence, or pregnancy (times of increased insulin resistance) or later in adulthood	Insulin or SU in the neonatal phase,Various glucose-lowering treatments in the relapse phase
SLC19A2-diabetes	● Diabetes onset is often in the infancy period, but can occur later in childhood or adolescence ● Megaloblastic anemia is responsive to treatment with thiamine ● Other main features include sensorineural deafness	Thiamine for anemia, in most cases insulin-treated

This systematic review is written on behalf of the American Diabetes Association (ADA)/European Association for the Study of Diabetes (EASD) *Precision Medicine in Diabetes Initiative* (PMDI) as part of a comprehensive evidence evaluation in support of the 2nd International Consensus Report on Precision Diabetes Medicine^7^. The PMDI was established in 2018 by the American Diabetes Association (ADA) in partnership with the European Association for the Study of Diabetes (EASD) to address the burgeoning need for better diabetes prevention and care through precision medicine^8^. The evidence for whom to test for monogenic diabetes, how to test them, and how to interpret a gene variant, as well as for underpinning the link between the genetic test result and prognostics are covered as separate systematic reviews in this series, by other members of the PMDI addressing precision diagnostics and prognostics for monogenic diabetes^9,10^.

We aimed to provide a systematic review of the evidence for precision medicine in beta-cell monogenic diabetes to answer several questions. What is the optimal glucose-lowering therapy in the three commonest subtypes of autosomal dominant familial diabetes also known as Maturity Onset Diabetes of the Young (MODY): GCK-related hyperglycemia, HNF1A-diabetes, and HNF4A-diabetes? What is the optimal glucose-lowering therapy in the two commonest subtypes of syndromic diabetes: HNF1B-diabetes and Mitochondrial Diabetes (MD) due to m.3243 A > G in the *MT-TL1* gene? Are there alternatives to insulin therapy in 6q24-related transient neonatal diabetes (6q24-TND); and does thiamine supplementation improve glycemia in SLC19A2-diabetes also known as Thiamine-Responsive Megaloblastic Anemia (TRMA) syndrome? While there is some support for no treatment in GCK-related hyperglycemia and sulphonylureas for HNF1A-diabetes, overall there is limited evidence to guide the treatment in monogenic diabetes with most studies being non-randomized and small.

## Methods

Protocols for systematic reviews were developed and registered in Prospero (https://www.crd.york.ac.uk/prospero/; for MODY (CRD42021279872); syndromic diabetes (CRD42021250955) and neonatal diabetes (CRD42023399408). The study was reported in accordance with Preferred Reporting Items for Systematic Reviews and Meta-Analysis (PRISMA) guidelines^11^.

### Search strategy

We comprehensively reviewed the literature associated with glycemic treatment outcomes, searching in PubMed, MEDLINE, and Embase separately for (1) GCK-related hyperglycemia, HNF1A-diabetes, and HNF4A-diabetes; (2a) HNF1B-diabetes; (2b) MD with m.3243 A > G variant; (3a) 6q24-TND; (3b) SLC19A2-diabetes. The Online Mendelian Inheritance in Man codes (and Orphanet rare disease nomenclature ORPHA codes) for the diseases are (1) #125851 (552), #600496 (552), #125850 (552), respectively; (2a) #137920 (552); (2b) #520000 (225); (3a) #601410 (99886), and (3b) #249270 (49827).

Filtering and selection of studies for full-text review and data extraction were recorded using Covidence (https://www.covidence.org). The search strategies and the PRISMA summaries for the searches are shown in Supplemental Tables 1–5 and Supplemental Figs. 1–4. At least two authors independently reviewed the titles and abstracts to filter out the articles for full-text review. Two authors independently reviewed full-text articles and either a third author (regarding GCK-related hyperglycemia, HNF1A-diabetes, and HNF4A-diabetes) or the two reviewers jointly (HNF1B-diabetes, MD, 6q24-TND, SLC19A2-diabetes) resolved discrepancies. One author for each search performed the data extraction using a standardized form and two reviewed it. Data were extracted from text, tables, or figures from the main and supplemental documents. Data extracted for each study included first author, publication year, country, study design, number of participants, age at diagnosis of diabetes and at the time of study, sex, diabetes duration, treatment information, and glycemic data.

### Inclusion and exclusion criteria

We included English language original articles (case reports, case series, cross-sectional studies, experimental studies, trials) written after 1992 (following the initial molecular characterization of monogenic causes of diabetes) concerning the treatment of hyperglycemia in human individuals diagnosed with monogenic diabetes subtypes of interest. Individuals with variants of unknown significance, multiple types of diabetes, and those lacking measures of treatment effect were excluded. Studies or data within studies that aggregated treatment effects for multiple monogenic diabetes types were also excluded. We included studies reporting on more than one monogenic diabetes type of interest with partially incomplete data, as long as data could be fully extracted for at least one monogenic diabetes subtype.

### Risk of bias and evidence appraisal

We used NHLBI study-quality assessment tools to assess the risk of bias (https://www.nhlbi.nih.gov/health-topics/study-quality-assessment-tools) and the methods outlined by Sherifali et al., to assess the level of evidence and to grade recommendations^12^.

### Data synthesis

Data were extracted from Covidence into Microsoft Excel. Data were summarized using Microsoft Excel. Data are presented as mean ± standard deviation, median [interquartile range, IQR], median (range), or as percentages.

Meta-analysis was not carried out due to the heterogeneity in study design as well as the high level of risk of bias in included studies, particularly case reports and series that make up a substantial portion of the literature.

### Reporting summary

Further information on research design is available in the Nature Portfolio Reporting Summary linked to this article.

## Results

### Risk of bias

Most studies across all diabetes types were rated as having moderate or serious risk of bias related to the study design (mainly comprising observational case reports and case series) and selection of the study population and outcome variables (with genetic diagnoses not based on non-targeted population screening). Whenever a response or a non-response to a drug was reported, it was unclear whether other factors significantly or moderately contributed to the reported response.

### What is the optimal glucose-lowering therapy in the three commonest subtypes of autosomal dominant familial diabetes (MODY): GCK-related hyperglycemia, HNF1A-diabetes, and HNF4A-diabetes?

There were 2389 unique studies identified by the literature search (Supplemental Fig. 1). After the title and abstract review, 136 studies remained for full-text review. Data was extracted from 33 articles in total, including six experimental studies (two designed as superiority trials), of which four were randomized controlled studies, 25 case reports or series, or cohort studies, one cross-sectional study, and one study presenting both cross-sectional and cohort data. There were 12 studies that contributed to data on treatment for GCK-related hyperglycemia, 23 studies for HNF1A-diabetes, and three studies for HNF4A-diabetes (with several studies contributing data to more than one diabetes subtype). The key summaries of these data are in Tables 2–5 and Supplemental Tables 6–7. The overall level of evidence was low and the risk of bias was high for most studies. The randomized controlled trials did not include power calculations.

**Table 2:** Experimental study of GCK-related hyperglycemia

Study name, design, and population	Primary endpoint	Key results(s)	Appraisal of study
Klupa et al.^14^ An observational cross-over study of 2 days of high, followed by 2 days of low-carbohydrate intake with a 1-day washout period 10 adults (4 females) with GCK-related hyperglycemia Mean age 37.4 (range 19–54) years (diabetes, *N* = 7), 30.0 (19–52) years (prediabetes, *N* = 3) Mean baseline HbA1c 7.3 (6.1–8.4)% (diabetes), 6.0 (5.9–6.1)% (prediabetes)	-Mean blood glucose level (MBG) -Percentage of time above target postprandial blood glucose level defined as 7.8 mmol/L	When the 3 patients with prediabetes were excluded, the remaining 7 patients had higher MBG during exposure to the high-carbohydrate diet than while on the low-carbohydrate diet (difference of 0.76 mmol/L, *P* = 0.02) They also had spent less time above the target postprandial blood glucose level (difference of 11.7%, *P* = 0.02)	-Lacked a control or comparison group -Did not control for carryover effects ● Short washout period ● Did not control for potential order effects ● All participants began with high- carbohydrate diet -Diet and daily caloric intake were not standardized -In analysis, groups were divided into diabetes and prediabetes-based on FBG. This resulted in overlap of HbA1c between groups and individuals with HbA1c values that are defined as prediabetes analyzed in the diabetes group. With study design limitations, short duration, and separate analysis of prediabetes from diabetes, which was not a predefined exclusion, no recommendation can be made on dietary treatment for GCK-related hyperglycemia.

**Table 3:** Experimental studies of HNF1A-diabetes

Study, design, and population	Randomized controlled superiority cross-over trial using intent-to-treat analysis and assessing for carryover effects of gliclazide or metformin (1-week washout period off treatment before/between treatments) in 18 adults (11 females) with HNF1A- diabetes (cases) matched by body-mass index and fasting plasma glucose (FPG) to 18 adults (6 females) with type 2 diabetes (T2D-controls); Pearson et al.^25^
Primary endpoint	Reduction in FPG
Key results	The cases had a 5.2-fold greater FPG reduction with gliclazide (4.7 mmol/L) than metformin (0.9 mmol/L) (no difference in T2D). The use of gliclazide led to a 3.9-fold greater fall in FPG in cases compared to T2D controls (no difference in metformin response).
Appraisal	Despite the small sample size and short duration, this study provides evidence to establish SU as a precision treatment for HNF1A- diabetes, showing treatment response differences by genotype.
Study, design, and population	Randomized, double-blinded, cross-over study comparing (after a washout period off treatment differing by diabetes regimen) the acute effect of nateglinide, glibenclamide, and placebo during a standard 450-kcal test meal and light bicycle exercise (at least 1-week washout between treatments) in 15 adults (10 females) with HNF1A-diabetes; Tuomi et al.^26^.
Primary endpoint	Prandial plasma glucose (PG) concentrations; Peak PG; Maximum increment in PG from fasting to 140 min; Incremental glucose area under the curve (AUC) in the first 140 min of the test
Key results	Peak PG and AUC were significantly lower at the nateglinide visit compared with glibenclamide or placebo. Six episodes of hypoglycemia occurred during exercise with glibenclamide compared to none with nateglinide and placebo (*P* = 0.030).
Appraisal	Although not a true treatment study, this is the only controlled study providing evidence for the use of glinides as an alternative to SUs when hypoglycemia is a concern and supports the limited case reports citing the beneficial use of glinides in these circumstances.
Study, design, and population	Randomized, double-blinded, placebo-controlled, cross-over study comparing 6 weeks of liraglutide and placebo to 6 weeks of glimepiride and placebo (1 week washout period off treatment before and between treatments) in 16 adults (8 females) with HNF1A-diabetes; Østoft et al.^27^.
Primary endpoint	FPG at baseline and at the end of each treatment
Key results	Both treatments lowered FPG significantly from baseline, but the decrement did not significantly differ (glimepiride −2.8 + /− 0.7 mmol/L; liraglutide −1.6 + /− 0.5 mmol/L). Of the 19 mild hypoglycemic events (in 11 participants), one occurred during treatment with liraglutide, and 18 during glimepiride
Appraisal	Despite the small sample size and short duration, this study provides evidence of the short-term efficacy of GLP1RA (liraglutide) in the treatment of HNF1A-diabetes. Larger studies with a longer duration allowing comparison of the HbA1c response are needed.
Study, design, and population	Randomized, double-blinded, placebo-controlled superiority cross-over study (with intent-to-treat analysis and assessment of carryover effects and the impact of subject relatedness) comparing 16 weeks on glimepiride and linagliptin to 16 weeks on glimepiride + placebo (4-week washout period between treatments) in 19 adults (11 females) with HNF1A- diabetes; Christensen et al.^28^.
Primary endpoint	Mean amplitude of glycemic excursions (MAGE) during 6 days of continuous glucose monitoring at the end of each treatment period
Key results	Reduction in MAGE did not differ significantly, but the use of glimepiride + linagliptin led to −0.5% [−0.9 to −0.2] lower HbA1c (*P* = 0.0048) and 15 vs. 32 episodes of hypoglycemia on CGM compared with glimepiride + placebo.
Appraisal	While the small sample size and relatively short duration are limitations, this study supports DPP4i (linagliptin) as an add-on therapy to SU. Frequently, the SU dose could be lowered by adding linagliptin and this was a weight-neutral approach compared to 1.2 kg weight gain with SU monotherapy.

**Table 4:** Summary of included studies for GCK-related hyperglycemia

Study ID	Number of participants	Sex (M/F)	Baseline group, therapy	Comparison therapy	Mean or median Pre-HbA1c (%)	Mean or median Post-HbA1c (%)	Mean, median, or minimal interval between pre/post HbA1c (months)
Cohort studies or case series							
Carlsson et al.^21^	29	17/12	20 None 7 Insulin 2 Metformin	29 None	6.3	6.2	64
Delvecchio et al.^22^	136	NR	133 None 2 Insulin 1 Insulin + metformin	133 None 2 Insulin 1 lost to follow-up	6.4	6.2	6
Shepherd et al.^23^	8	NR	7 Insulin 1 Metformin	8 No	6.6	6.6	15
Stride et al. ^24^	18 10	NR NR	None Insulin	None None	6.4 6.5	6.4 6.3	3 3
	6	NR	Oral Hypoglycemic agents	None	6.4	6.3	3
Cross-sectional studies (uncontrolled group comparisons)							
Stride et al.^24^	799	NR	Insulin therapy (*n* = 60) Median HbA1c (%) 6.3 [6.0, 6.6]	Oral hypoglycemic agent therapy (*n* = 108) Median HbA1c (%) 6.5 [6.1, 6.9]	No pharmacologic therapy (*n* = 631) Median HbA1c (%) 6.4 [6.1, 6.7]

**Table 5:** Summary of included studies for HNF1A- and HNF4A-diabetes

Study ID	Number of participants (HNF1A/HNF4A)	Sex (M/F)	Baseline group treatment	Comparison	Mean or median Pre-HbA1c (%)	Mean or median Post-HbA1c (%)	Mean, median, or minimal interval between pre/post HbA1c (months)	
HNF1A-diabetes & HNF4A-diabetes Cohort studies or case series								
Carlsson et al.^21^	17 (10/7)	3/7 (HNF1A) 1/6 (HNF4A)	5 None 10 Insulin 1 Insulin + MET 1 MET	5 None 3 Insulin 7 SU, SU + insulin 1 SU + DPP4i	8.3	7.0	83	
Shepherd^22,23^	13 (11/2) (Median diabetes duration: 4.6)	9/4	12 Insulin 0 Insulin + MET 1 MET	1 Diet 12 SU	7.5	6.4	24	
	23 (18/5) Median diabetes duration: 18.1	19/4	17 Insulin 2 Insulin + MET 4 MET	2 Diet 6 SU, 6 SU + MET, 3 SU + insulin, 3 SU + insulin + additional agent, 3 Insulin ± non-SU additional agent	8.8	9.2	24	
HNF1A-diabetes Cohort studies or case series								
Kyithar et al.^44^	4 2	NR NR	None Insulin	SU SU	7.5 7.4	6.8 6.5	16 19	
HNF1A-diabetes uncontrolled experimental study								
Shepherd et al.^29^	8	NR	Insulin	SU	Median reduction in HbA1c (%)0.8 (−2.5–3.2)	6		
HNF1A-diabetes Cross-sectional studies (uncontrolled group comparisons)								
Raile et al.^45^	114^a^	41/73	Mean HbA1c (%)					
			Insulin (*n* = 34)	SU (*n* = 16)	Insulin + SU (*n* = 14)	Glinides (*n* = 13)	Glinides + Insulin (*n* = 9)	None (*n* = 28)
			7.5 [6.2, 8.3]	6.7 [5.8, 7.4]	7.9 [6.7, 8.9]	6.8 [5.9, 7.2]	7.2 [6.6, 7.9]	6.1 [5.1, 6.6]

*SU* sulfonylurea, *MET* metformin, *DPP4i* DPP4 inhibitor.

^a^There were no statistically significant differences in age, diabetes duration, or BMI between treatment groups.

### What is the optimal glucose-lowering therapy in GCK-related hyperglycemia?

We originally posed the question of what is the impact of pharmacological and non-pharmacological glucose-lowering therapy in GCK-related hyperglycemia. However, we identified only one case report that presented data on active pharmacological intervention^13^ (Supplemental Table 6) and one study on dietary intervention^14^ (Table 2), and the rest of the 10 studies^15–24^ (Table 4 and Supplemental Table 6) assessed the impact of stopping anti-hyperglycemic agents on HbA1c or glucose or assessed the stability of HbA1c over time on no therapy. Thus, we concentrated on analyzing the evidence to support or refute non-treatment of GCK-related hyperglycemia.

Most studies on GCK-related hyperglycemia looked at within individual stability of HbA1c over time without glucose-lowering therapy (175 individuals from case reports and cohort data, range of time between 3–126 months)^15,17,19,21,22,24^ or after cessation of pharmacologic therapy following genetic diagnosis (35 individuals from case reports and cohort data)^16,20,21,23,24^. All studies showed stability of HbA1c with no significant change including when glucose-lowering therapy of either oral hypoglycemic agents (OHA) or insulin was discontinued (Table 4, Supplemental Table 6). Only a single case report suggested adding therapy might lower HbA1c^13^ (Supplemental Table 6).

A single large cross-sectional cohort study (*n* = 799) showed no differences in HbA1c between observational non-randomized treated and untreated groups^24^ (Table 4).

There were no randomized long-term treatment trials in GCK-related hyperglycemia. We identified a single experimental cross-over study for GCK carriers^14^ that assessed the impact of high-carbohydrate (60%) vs. low-carbohydrate (25%) unstandardized diet on mean glucose levels (MBG) and time spent above target postprandial blood glucose level (7.8 mmol/L) as measured by continuous glucose monitor (CGM) in 10 GCK subjects (Table 2). The duration of each intervention was brief (2 days with 1 day washout period). A statistically significant difference in mean glucose level (0.78 mmol/L) and time above target (11.7%) was found after high-carbohydrate diet if the analysis was restricted to the seven patients with an initial HbA1c above 6.5% (a non-predefined analysis). No comparison groups such as individuals without diabetes or with type 2 diabetes were included.

### What is the optimal glucose-lowering therapy in HNF1A-diabetes and HNF4A-diabetes?

We analyzed studies for evidence of SU as an effective glucose-lowering therapy for HNF1A-diabetes and HNF4A-diabetes. We also assessed evidence for alternative or augmentative non-insulin therapies.

A striking observation was that all the trials or experimental studies (4 trials, 1 uncontrolled study, 76 individuals)^25–29^, and almost all the observational data (18 studies, 182 individuals)^21,23,30–45^ were for HNF1A-diabetes. While five studies included both HNF1A-diabetes and HNF4A-diabetes, only three studies (16 individuals) had HNF4A-diabetes data that could be extracted^21,22,34^.

In HNF1A-diabetes a single superiority, randomized cross-over study tested using SU in comparison with type 2 diabetes^25^ (Table 3). This was the only study in monogenic beta-cell diabetes to have a comparative group with type 2 diabetes, hence truly testing if it was a specific precision approach. The study compared the fasting plasma glucose (FPG) response in individuals with HNF1A-diabetes (*N* = 18) and type 2 diabetes (*N* = 18) to therapy with SU (gliclazide) or metformin for 6 weeks. The study groups were matched for body-mass index and FPG after the wash-out period off-treatment but not for sex or age. Gliclazide was superior to metformin in lowering fasting glucose in HNF1A-diabetes but not in type 2 diabetes (5-fold greater response to SU than metformin in HNF1A-diabetes; no difference in type 2 diabetes).

Glinides have been proposed as an alternative to long-acting SU, for example in instances of problematic hypoglycemia. There was one randomized, placebo-controlled cross-over study that compared the acute effects of premeal dosing of SU (1.25 mg glibenclamide), glinide (30 mg nateglinide) or placebo on glucose excursions during and after a standardized meal and exercise in 15 participants^26^ (Table 3). When comparing glibenclamide and nateglinide response to the standardized meal, peak insulin occurred earlier and plasma glucose levels (peak and up to 140 min) were lower with nateglinide. Exercise after the meal resulted in hypoglycemia (glucose <3.5 mmol/L) in 6 of 15 participants after glibenclamide while there were no episodes of hypoglycemia after nateglinide. Among the case studies, nine individuals from six studies initiating SU or glinide therapy showed an average HbA1c decrement of 1.3% after an average of 12.2 months of treatment^31,34–36,40,44^ (Supplemental Table 7).

We analyzed whether HNF1A patients treated with insulin therapy can successfully transfer to SU. In a prospective study on HNF1A-diabetes (*n* = 27) and HNF4A-diabetes (*n* = 7) not on SU at diagnosis, 25 of the 31 patients on insulin discontinued insulin but only 12 (48%) achieved an HbA1c of 7.5%^23^ (Table 5). Good glycemia was associated with a shorter diabetes duration and lower HbA1c at transfer. An uncontrolled study of eight individuals with HNF1A-diabetes assessed the success of transitioning from insulin that had been used since diabetes diagnosis (median insulin dose 0.5 units per kg; median duration of insulin treatment 20 years) to SU (gliclazide, median dose 80 mg daily)^29^ (Table 5). All patients were able to discontinue insulin. The HbA1c response was variable with a median reduction of 0.8% and six of eight improving but one individual had a worsening of HbA1c by 3.2%. Case-level data also show that seven individuals from five studies transitioning from insulin to SU showed an average HbA1c decrement of 1.35% after an average of 6.2 months of treatment with SU^33,34,38,42,44^ (Supplemental Table 7).

Two randomized, double-blinded cross-over studies compared alternative and augmentative regimens to SU monotherapy in HNF1A-diabetes (Table 3). Sixteen patients were enrolled in a trial comparing the change in FPG and risk of hypoglycemia during 6 weeks of SU monotherapy (glimepiride) and 6 weeks of glucagon-like peptide-1 receptor agonist (GLP1RA) monotherapy (liraglutide) with a 1-week washout between medications^27^. Among the 15 patients who completed the trial, FPG and post-prandial glucose were lowered by both treatments without a statistically significant difference between them. The number of mild hypoglycemic episodes (glucose <3.9 mmol/L) was markedly higher with glimepiride therapy (18 events) compared to liraglutide (1 event). The second superiority trial compared the effects of 16 weeks of SU monotherapy (glimepiride) to 16 weeks of combination therapy with glimepiride and the dipeptidyl peptidase-4 inhibitor (DPP4i) linagliptin (with 4-week washout) on the mean amplitude of glycemic excursions (MAGE) measured by CGM^28^. The combination therapy did not have an impact on MAGE over that of SU alone, but the mean (95% CI) HbA1c showed a significant decrease by −0.5% (−0.9 to −0.2, *P* = 0.0048) between SU and the combination therapy, included as a secondary endpoint.

There were only three studies where HNF4A data were reported separately^21,23,34^ (Table 5, Supplemental Table 7). Globa et al., report on two individuals with HNF4A-diabetes showing good response to SU, with >1% decrease in HbA1c (one was treatment-naive, the other switched from metformin)^34^. Another study included seven children with HNF4A-diabetes. Only two were on SU alone (mean HbA1c 7.0%, mean follow-up 8.3 years), while three were treated with insulin, two by choice and one due to inadequate control after switching from insulin to SU. Two others were not on pharmacologic treatment^21^.

### What is the optimal glucose-lowering therapy in the two commonest subtypes of syndromic diabetes due to pathogenic variants in HNF1B and mitochondrial diabetes

After duplicate removal and abstract screening of 1716 articles, 135 articles remained for full-text review. Data was extracted from 18 articles for HNF1B-diabetes, 42 for MD, and 2 for both (Supplemental Fig. 2). We manually added an article not identified in the search^46^. There were no controlled trials, the overall level of evidence was low, and the risk of bias was high. Tables 6 and 7 show a summary of the data for the 13 articles on HNF1B-diabetes^46–58^ and 10 articles on MD^46,59–67^ that reported any treatment response even in single cases, and the prevalent treatment modalities in patient cohorts. The articles represent altogether 293 cases with HNF1B-diabetes and 242 with m.3243 A > G MD. Most articles only stated the current medication, which precludes drawing conclusions on the efficacy, but we note that at least one-fifth of the patients in both groups had no insulin treatment.

**Table 6:** Summary of included studies for HNF1B-diabetes

HNF1B-diabetes Cohort studies or case series (*N* = 293 (132 men, 161 women)										
**Study ID**	**Number of participants**	**Sex (M/F)**	**Variant (intra-genic/ deletion)**	**Age (years)**		**Duration of diabetes (years)**	**Treatment before/after the genetic diagnosis**		**Intervention**	**Response**
				**Diagnosis**	**Last appraisal**		**Before**	**After**		
Dubois- Laforgue et al.^47^ *Adults*	159	73/86	75/84	28 [20–37]	45 [3–56]	12 [5.5–22.5]	68 INS 47 OAD 25 LS^a^	111 INS 22 OAD 7 LS	SU or repaglinide (*n* = 51^b^)	29 responders (57%): HbA1c 7.1% [5.5– 12.1] → 6.1% [4.4–7.0], *p* < 10^−4^, during treatment for 5 [3–9] years
									INS replaced with SU (*n* = 10)	Three could convert
^c^Colclough et al.^46^	50	26/24	26/24	18 [13–27]		3 [1–8]	23 INS 4 INS + OAD			
	18	7/11	4/14	17 [12–25]		3.5 [1–9]	10 INS 4 INS + OAD			
Warncke et al.^48^ *Minors*	35	16/19	NA mostly deletions	13.5 [11.2–15.7]	13.8 [12.4–16.3]	<1	20 INS 3 INS + OAD 4 OAD, 8 LS			
Ng et al.^49^	10	6/4	9/1	31.5 [16–39]	55 (18–62)	18.5 (4–47)	4 INS, 2 INS + OAD 2 OAD 1 LS	6 INS 2 INS + OAD 1 OAD (MET)	Trial of SU for 5 patients on INS	none weaned off INS
Kettunen et al.^50^	11	2/9	4/7					9 INS 2 LS		
Case reports combined										
^ d^(see foot-notes)	10	2/8	5/5	16.5 [15.8–18.3] (*N* = 8)	22.5 [18.3–33.5]	3 (0–22)	5 INS 1 OAD (MET)	3 INS 1 INS + OAD 2 OAD (MET) 1 GLPRA 1 LS	Duration of non-INS response (HbA1c < 6.5–7%): at least 1 year on GLP1RA (Terakawa) 6 years on SU+DPP4i (Carrillo) at least 1 year on MET (Tao, Thirumalai)	

Data are shown as median (range) or [interquartile range] or mean ± SD; *M/F* number of men/women, *NA* not available, *INS* insulin, *OAD* oral antidiabetic agent, SU sulphonylurea, *MET* metformin, *GLP1RA* GLP1-receptor agonist, *DPP4i* DPP4 inhibitor, *TZD* thiazolidinedione, *AGI* Alpha-glucosidase inhibitor.

^a^At diagnosis.

^b^These were included among the cohort of 159 patients (diabetes duration 0.75 [0–5.25] years), with response defined as HbA1c < 7%.

^c^Genetic testing laboratory serving clients globally; two subcohorts based on clinical suspicion of HNF1B disease (*N* = 50) or MODY (*N* = 18).

^d^Roehlen (2018), Terakawa (2020), Carrillo (2017), Tao (2020), Aydın (2022), Ren (2021), Thirumalai (2013), Mateus (2020), with PMIDs: 30032214, 32871938, 28680642, 32756155, 35899569, NA (10.1007/s13410-020-00904-6), 23480312, 32864159.

**Table 7:** Summary of included studies for mitochondrial diabetes

Mitochondrial diabetes caused by m.3243 A > G Cohort studies or case series (*N* = 242 (91 men, 146 women, NA: 5), see also the footnote)									
Study ID	Number of participants	Sex (M/F)	Age (years)		Duration of diabetes (years)	Treatment before/after the genetic diagnosis		Intervention	Response
			At diagnosis	At appraisal		Before	After		
Guillausseau et al.^59^	77	31/46	38.8 ± 9.6 (12–67)	48.6 ± 10.2 (31–71)	11.0 ± 9.0 (0–37)	13 INS 64 non- INS	44 INS 21 SU and/or MET 12 LS		
Esterhuizen et al.^60^	30	9/21		50.0 ± 9.6			22 INS 2 MET 10 SU (partial overlap)		
	5^a^						3 INS, 1 OAD		
^b^Colclough et al.^46^	54	22/32	33.5 [26–39]		6 [2–15]	34 INS 8 INS + OAD			
	24	6/18	28 [22–34.5]		2 [1–7.5)	13 INS 2 INS + OAD			
^c^Suzuki et al.^61^	28	13/15	38.7 ± 10.2	43.5 ± 12.3			14 INS, 8 OAD, 6 LS	(Coenzyme Q10)	C-peptide; no glycemic endpoints
	16	8/8	39.6 ± 8.7	44.4 ± 9.8			8 INS, 4 OAD, 4 LS	(non-blinded control group)	
Case reports combined									
^d^(see foot-notes)	8	2/6		47.5 (32–72)	0–30	2 MET;1 AGI; 1 DPP4i+MET	1 INS; 2 GLP1RA; 1 SGLT2i; 2 DPP4i 1 SGLT2i+D PP4i; 1 TDD		

Data are shown as median (range) or [interquartile range] or mean ± SD; *M/F* M/F, number of male/female cases,*NA* not available, *INS* insulin, *OAD* oral antidiabetic agent, *SU* sulphonylurea, *MET* metformin, *GLP1RA* GLP1-receptor agonist, *DPP4i* DPP4 inhibitor, *TDD* thiazolidinedione, *AGI* Alpha-glucosidase inhibitor.

^a^5 patients with diabetes and mitochondrial encephalomyopathy, lactic acidosis, and stroke-like episodes (MELAS), not included in the 30 patients above.

^b^Genetic testing laboratory serving clients globally; two subcohorts based on clinical suspicion of mitochondrial diabetes (*N* = 54) or MODY (*N* = 24).

^c^A larger cohort described by Suzuki et al. in 2003 (PMID 12590018) included 113 patients with MD, of whom 86.1% required insulin. Because of potentially overlapping data, the table includes this open trial in 1998 with more detailed clinical data. To calculate the proportion of patients with MD on insulin, we used the larger cohort instead of the cohort in the table and excluded Colclough (2022) with no treatment data after the genetic diagnosis (resulting in a total cohort of *N* = 233).

^d^Lebbar (2021), Cosentino (2019), Keidai (2019), Ninomiya (2016), Lin (2022), Yeung (2021).

There were no randomized studies or trials of treatment in HNF1B-diabetes. Of the 168 individuals with HNF1B-diabetes, for whom treatment data after the genetic diagnosis was available, 132 (79%) used insulin, but it is not known if other medications had been tested (Table 6). One French study systematically evaluated the use of SU or repaglinide after the genetic diagnosis at a median [IQR] duration of 0.75 [0–5.25] years and reported that 29 of 51 (57%) patients displayed an HbA1c decrease. However, the duration of SU or repaglinide treatment in those who did respond was 5 [3–9] years, and at a mean of 12 years follow-up, 79% of the cohort were on insulin^47^. They also tried replacing insulin with SU for 10 patients, which was successful in three (no details given). In an uncontrolled Irish study, none of five patients on insulin could successfully switch to sulfonylureas^49^.

There were no randomized studies or trials of treatment in m.3243 MD. Of the 233 individuals with MD, for whom treatment data after the genetic diagnosis was available, 167 (72%) used insulin, but again it is not known if other medications had been tested (Table 7). No studies are reported trying to discontinue insulin by treating with OHAs.

Evidence against the use of metformin was limited to three case reports^66,68,69^ (Table 7). In two of them, metformin use was associated with elevated lactate levels (2.5–3.7 mmol/L^68^, and up to 5.9 mmol/L^66^), and the first was reported to have lactic acidosis. However, pH was not given for either case, and an improved lactate level (2.4 mmol/L) after discontinuation of metformin was only given for the latter case.

### Are there alternatives to insulin therapy in 6q24 transient neonatal diabetes (6q24-TND)?

The literature search identified 1489 studies related to 6q24-TND (Supplemental Fig. 3). After duplicate removal, abstract screening, and full-text review, 19 studies met eligibility criteria, including five case series reporting on 16 cases, 14 reports of single cases, for a total of 30 6q24-TND cases for whom data was available regarding treatment with non-insulin therapies (Supplemental Tables 8 and 9).

There were no randomized trials for therapeutic response in 6q24-TND. For 16 cases with relevant data on the initial neonatal phase of diabetes^70–79^(Supplemental Table 8), SU (glyburide or glibenclamide in nearly all cases) was the only class of medication used other than insulin. Efficacy of SU during the neonatal phase was inconsistent, with studies reporting no effect or failure of SU to improve diabetes management in seven cases^72,74–76,78,79^, while for nine cases SU treatment was reported to allow insulin to be discontinued^70,71,73,77–79^. Of note, in most cases, diabetes remitted within days to weeks after insulin was discontinued, but three cases were reported to remain insulin-treated as old as 41- 60 months of age at the time of the reports^72,74,78^. None of these three cases with a possibly more permanent neonatal diabetes phenotype exhibited a response to SU treatment.

For case reports with relevant data on the use of non-insulin therapies during the later relapse phase of diabetes^79–88^ (Supplemental Table 9), the apparent efficacy of such treatment was more consistent, with 13 of 14 cases being either able to discontinue insulin at least temporarily or were never started on insulin (three cases)^79–86,88^. There was a wide range of follow-up time after the initiation of non-insulin therapies, but in most cases, it was at least 6 months and in a few cases many years. Measures of glycemia were reported inconsistently. The most common class of medications utilized was SU (most commonly glyburide or glibenclamide), with some reporting the use of metformin (either as monotherapy or as an adjunctive agent), and a few cases utilized DPP4i (either alone or with SU). For the one patient who was not able to discontinue insulin, metformin was the only additional agent utilized^87^. The only adverse events reported were rare mild gastrointestinal symptoms and mild hypoglycemia, but these may have been underreported.

### Does thiamine supplementation improve glycemia in SLC19A2-diabetes, also known as Thiamine-Responsive Megaloblastic Anemia Syndrome (TRMA)?

The literature search yielded 166 studies (Supplemental Fig. 4). After duplicate removal, abstract screening, and full-text review, 32 studies were included in the review with three larger case series and 29 case reports. Varying data on the effect of thiamine therapy on SLC19A2-diabetes were available for 95 patients (at the individual level, *n* = 72 patients; at group level *n* = 23 in one case series) (Table 8, Supplemental Table 10, and Supplementary Data 1). There were no randomized trials for therapeutic response in SLC19A2-diabetes, and the scope of the studies was mainly on the overall description of the phenotype.

**Table 8:** Summary of included larger case series studies for SLC19A2-diabetes (TRMA syndrome)

SLC19A2-diabetes, case series	Number of patients, *n* (males, %)	Age at diabetes diagnosis, median (years)	Age at Thiamine start, median (years)	Thiamine dose (mg/day), median	Current age, median (years)	HbA1c (%), median		Outcome, if specified	Clear response to Thiamine Yes, *n* (%)
						Pre	Post, latest		
Habeb et al.^118^	15 (NR)^a^	1.4 (Range, 0.1–3.5)	2.0 (IQR, 1.0–7.4)	NR (Range Max, 25–300)	14 (Range, 3.7–31.5)	9.0 (Range, 6.8–11.9)	7.8 (Range, 5.2–9.1)	27 % insulin-independent, *n* = 4	11 (73%)^b^
								47% Reduced dose or HbA1c, *n* = 7	
								27% No response, *n* = 4	
Ricketts et al.^119^	13 (38%)^c^	2.2 (Range, 0.5–12)	NR	50 (Range, 25–150)	9 (Range, 2–30)	NR	NR	15 % insulin-independent, *n* = 2	NR
Warncke et al.^120^	23 (52%)^d^	1.4 (IQR, 0.8–3.6)	5.9 (IQR, 2.4–12.4)	Initial, 100 (IQR 80–200)	14.3 (IQR, 8.1‒17.5)	NR	6.9 (IQR, 6.1‒7.9)	13% Insulin-independent, *n* = 3	NR
				Current, 200 (IQR, 100–300)					

^a^Individual follow-up data of the total 32 patients of the study. Includes follow-up data of seven patients previously reported. .

^b^Clear response was defined when there was a decrease in daily insulin dose of ≥10% or in HbA1c of ≥1%.

^c^Includes follow-up data of six patients previously reported. .8105295).

^d^Individual data not available. *n* = 18 (78%) of the patients had genetic confirmation of TRMA.

Diabetes was diagnosed at the median age of 1.15 (range, 0.2–5.4, *n* = 44) years in case reports^89–117^ (Supplemental Table 10 and Supplementary Data 1) and between median ages of 1.4 and 2.2 (0.1–12, *n* = 51) years in the three case series^118–120^ (Table 8). Insulin was the most common initial therapy for diabetes (*n* = 89/95) with one patient also using SU (glimepiride), and 6 not receiving any antidiabetic therapy prior to thiamine therapy. Thiamine therapy was initiated after a median duration of diabetes of 4 months (0–17.9 years, *n* = 53/95 with available data), two additional patients were already on thiamine therapy at the time of diabetes onset.

The median thiamine treatment duration at the time of follow-up was 0.9 years (2 days–25 years, *n* = 39) in case reports, 4.7 years (2–17.5 years, *n* = 15) in one case series, and not available in the remaining patients (*n* = 41). The initial and maximum median thiamine doses were 100 (25–200) and 100 (25–600) mg/day, respectively (*n* = 41), adjusted according to the response to anemia.

The effect of thiamine treatment on glycemic control was inconsistently reported using different outcome measures. Regarding the 72 patients with individual data, 8% (*n* = 6) remained insulin-independent from diabetes onset until the median follow-up time of 1 year (3 days–3 years) after initiation of thiamine. Among those on insulin, the commencement of thiamine treatment was associated with: achievement of insulin-independency in 24% (*n* = 17), a decrease in daily insulin requirement in 38% (*n* = 27), improvement in glycemic control without specification in 4% (*n* = 3), no response defined as unchanged insulin requirement or glycemic control in 26% (*n* = 19). Three patients required insulin treatment again at puberty. Only 11 of all 95 patients had reached adulthood (aged >18 years) –all of these were on insulin therapy.

Glycemic control both prior to and after the initiation of thiamine treatment was reported for 29 of 95 (31%) patients with a median pre-HbA1c of 8.7% (5.9–21.0%) and median post-HbA1c 6.7% (5.2–9.1%)^90,94,95,101–104,106,110,113–116,118^. Notably, the reported pre-HbA1c was in most cases measured at diabetes onset, therefore a decrease in HbA1c does not alone reflect the thiamine effect on glycemic control, but could also reflect the effect of the standard treatment of diabetes. Insulin dose at both timepoints was available for 16 of 89 (18%) insulin-treated patients, with the median dose of 0.68 (0.42–1.8) IU/kg/day prior to thiamine treatment and 0.4 (0.0–0.8) IU/kg/day while on thiamine treatment.

The initial and maximum median thiamine doses were 100 (25–200) mg/day and 100 (25–600), respectively, mg/day (*n* = 41), adjusted according to the response to anemia.

## Discussion

To the best of our knowledge, this is the first systematic review to look at the evidence for precision treatment of beta-cell monogenic diabetes outside of K_ATP_ neonatal diabetes, in which SU was previously shown to be an effective treatment with the success conditioned by differences in pharmacogenetics, age, pharmacokinetics, compliance, and maximal dose used^5^. Monogenic diabetes is an excellent candidate for precision medicine as the genetic etiology identified by genetic testing defines a subtype of diabetes with a specific pathophysiology enhancing the likelihood for a specific therapy to be most effective. For several forms of beta-cell monogenic diabetes, the underlying pathoetiology provides a rationale for precision treatment, but it is important to assess to what extent these rationales are supported by published evidence.

We sought to assess the evidence base for current treatment recommendations for several more common forms of beta-cell monogenic diabetes. Overall we found that there is limited, and mostly poor-quality evidence, mainly consisting of descriptive case reports and case series. There were limited randomized studies providing stronger evidence, some with considerable effect sizes, but limited by small numbers of participants and short durations resulting in inadequate power to detect or exclude differences. The majority of included cases are of European descent. Future efforts in this field should include racial and ethnic groups that have been underrepresented in the study of monogenic diabetes. The strongest evidence for a precision approach was, in order, HNF1A-diabetes, GCK-related hyperglycemia, relapse of 6q24-TND, and SLC19A2-diabetes. The evidence for insulin or non-insulin therapies in MD and HNF1B-diabetes was not clear and there was almost no evidence for HNF4A-diabetes treatment. Each subtype is discussed below.

### GCK-related hyperglycemia

The aggregate of data suggests that glucose-lowering treatment should not be given in GCK-related hyperglycemia. HbA1c stays stable in target range without initiating or after cessation of medical treatment and there is no evidence to support treatment for lowering glycemia. However, diagnostic testing for GCK is often guided by recruiting individuals with mild hyperglycemia and this may introduce bias to the phenotype seen. Against this, in non-selected sequencing in the general population^121^ and type 2 diabetes^122^, a mild glycemic phenotype was seen in subjects with *GCK* variants.

There was no evidence to support dietary recommendations specific to GCK-related hyperglycemia. Further work is needed on whether a dietary approach needs to be any different from the general population.

Other important topics for assessment in GCK-related hyperglycemia in the future were identified. While GCK-related hyperglycemia occurring in isolation does not need pharmacologic treatment, it is important to develop clinical recommendations for diagnosing and treating type 2 diabetes co-occurring with GCK-related hyperglycemia. Additionally, the long-term cardiovascular effects of mild hyperglycemia in GCK-related hyperglycemia need to be established in an older population.

A limitation of this systematic review is that we did not address the evidence for a precision medicine approach in pregnancies affected by maternal beta-cell monogenic diabetes. This is an important area, particularly in GCK-related hyperglycemia, where treatment may be required for pregnancies in women with GCK-related hyperglycemia carrying non-affected fetuses^123^. Management guidelines exist but are debated. Additionally, the potential impact of cell-free fetal DNA to provide early genotype information to guide maternal insulin therapy will need to be studied both in terms of treatment impact and cost-effectiveness.

Recommendation: No medical treatment should be given in GCK-related hyperglycemia (grade C evidence). No recommendation can be given regarding dietary treatment in GCK-related hyperglycemia.

### HNF1A-diabetes and HNF4A-diabetes

With the available data, we can only provide assessment of the evidence in HNF1A-diabetes although combined cohorts suggest results may be similar in HNF4A-diabetes. Clearly, more treatment studies are required in HNF4A-diabetes.

The strongest evidence in this systematic review for precision therapy was for the use of SU in HNF1A-diabetes. The key evidence came from a randomized cross-over study of SU and metformin therapy in HNF1A-diabetes and matched subjects with type 2 diabetes^25^ and was additionally supported by cohort and case studies demonstrating the initial efficacy of SU at diabetes diagnosis and the possibility of transitioning from insulin therapy after a genetic diagnosis is made, especially when close to diagnosis and when the HbA1c is well controlled^23^. While SU are a clear representation of diabetes precision medicine, they are non-efficacious in some individuals and their efficacy may not be durable in others, with diabetes duration and weight gain being two factors associated with reduced SU efficacy. Further studies are needed to elucidate factors that influence the response to SU within HNF1A-diabetes.

While there is support for the effectiveness of glinides in HNF1A-diabetes as an alternative to SU, particularly to address hypoglycemia^2631,45^, and for the newer diabetes medications as monotherapy (GLP1RA)^27,30,43^ or augmentative therapy (DPP4i)^21,28,32,34,37,39^, studies are of short duration and limited in number. There are no studies on drug-naïve newly-diagnosed patients with HNF1A- or HNF4A-diabetes comparing the different treatment options. This would be needed to conclusively suggest first-line (or second-line) treatment of hyperglycemia. The increased cardiovascular risk associated with HNF1A-diabetes provides another reason that carefully designed larger, long-term comparison studies of glycemic and cardiovascular outcomes of these newer classes of diabetes medications, which offer cardiovascular benefit, are needed^124–126^. On the other hand, it is not clear whether SGLT2i can be safely used in HNF1A-diabetes, which features insulin deficiency and already reduced expression of SGLT2 and glucosuria.

Recommendation: SU should be used as first-line therapy for HNF1A-diabetes (grade C evidence). Glinides can be used if frequent hypoglycemia is experienced with SU treatment (grade D evidence). GLP1RA is an option in the treatment of HNF1A-diabetes (grade C evidence). DPP4i are an option in the augmentative treatment of HNF1A-diabetes (grade C evidence). No recommendation can be given for HNF4-diabetes.

### HNF1B-diabetes and Mitochondrial diabetes

There is no defined precision treatment approach or even clear first-line medication for MD or HNF1B-diabetes, with the evidence for specific treatment in HNF1B-diabetes and MD being of low quality with no trials and very few studies. The degree of insulin deficiency among individuals with HNF1B-diabetes and MD can range from mild hyperglycemia to absolute insulin deficiency. While insulin has been advocated as the choice of treatment for both HNF1B-diabetes and MD^4,127^, we found no systematic evidence favoring the use of insulin. There are no RCTs on HNF1B-diabetes or MD and even open treatment studies and cohort or case reports are few. Thus, the results of the systematic search remain descriptive, reflecting the choices of the treating physicians. In the included case reports and case series, which were mainly cross-sectional, most patients diagnosed with HNF1B-diabetes or MD seem to have commenced insulin treatment at some stage. Besides insulin deficiency and secondary failure of other medications, this could be affected by a diagnostic selection bias or a progressive kidney disease precluding many modes of treatment. Similarly, there is no evidence for using or avoiding any of the other diabetes medications. We note that metformin, SU or glinides, DPP4i, SGLT2i, and GLP1RA were used in the case reports and case series, especially close to diagnosis. There is a rationale for studying SGLT2i specifically in HNF1B-diabetes, as it might improve the renal outcome of the associated non-diabetes-related kidney disease. However, the marked insulin deficiency often seen in HNF1B-diabetes could markedly increase the risk of diabetic ketoacidosis. Further large systematic and controlled studies are required in both HNF1B-diabetes and MD.

Lactatemia and/or lactic acidosis are part of the syndrome caused by the *MT-TL1* m.3243 A > G variant. Thus, avoidance of metformin has been recommended in MD as it impairs mitochondrial function and may further increase the risk of lactic acidosis^4^. However, the evidence against its clinical use was low and limited to three case reports with adverse effects, including only one patient with possible lactic acidosis^68^ and another with an increase of lactate levels possibly associated with metformin use^66^. MD needs additional preclinical and clinical studies to define the risk of metformin and statin use to guide therapy approaches. Patients with *MT-TL1* m.3243 A > G variant and associated neurological disease might be on coenzyme Q10 or other dietary supplements, like arginine, in an attempt to support their mitochondrial function but most studies have not examined the effects of the supplements on glucose control.

Recommendation: There is insufficient data to recommend or refute the preferential use of insulin or any other medication in HNF1B-diabetes or mitochondrial diabetes. However, in case of patients not on insulin therapy, the potential deterioration of insulin secretion should be evaluated if glucose control deteriorates.

### 6q24 transient neonatal diabetes

The evidence guiding the use of non-insulin therapies for 6q24-related diabetes is of low quality and more information is needed to make firm conclusions. In the initial neonatal phase of 6q24-TND, SU use was not always successful in improving diabetes outcomes or allowing for cessation of insulin. The evidence for the efficacy of non-insulin therapies was stronger for the relapse phase of diabetes later in life, where most cases appeared to benefit from a variety of non-insulin therapies, with most not requiring insulin. There is a pressing need to improve the evidence base for the management of 6q24-TND in both the neonatal and relapse phases and for long-term outcome data.

Recommendation: There is insufficient data to recommend or refute the preferential use of non-insulin therapies for the treatment of the neonatal or relapse phase of 6q24-TND. However, non-insulin therapy seems beneficial in patients where diabetes recurs, but we recommend close follow-up to ensure treatment intensification if patients do not reach or remain stable at their glycemic target (grade D evidence).

### SLC19A2-diabetes (TRMA)

The evidence for the use of thiamine improving glycemic control in SLC19A2-diabetes is of low quality because it is limited to case reports and series that provide inconsistent data on outcomes. However, there is a trend across the studies reporting thiamine administration resulting either in a reduction in insulin dose or allowing for discontinuation of insulin therapy, with stable or improved glycemic control, in a majority of the affected individuals. Quantifying an improvement in endogenous insulin secretion in patients treated with insulin is difficult but a common assessment is the daily insulin dose per kg in patients who maintain a similar level of glycemia. Further trials assessing this in the short and long term (especially beyond puberty) are required to firmly document the effect of thiamine on glycemic control. Although most cases not controlled by thiamine treatment alone were treated with insulin, further study could reveal the utility of treatment with other diabetes medications. This special situation and the rarity of the disease make clinical trials difficult to conduct.

Recommendation: Whereas thiamine treatment is essential for all patients with anemia in SLC19A2-diabetes,

the evidence supporting a specific and sustained effect of thiamine on glycemic control in SLC19A2-diabetes is

weak. We recommend thiamine be started as soon as a diagnosis of SLC19A2-diabetes is made. Additionally, we recommend close follow-up of patients with SLC19A2-diabetes after the initiation of thiamine treatment to adjust the diabetes treatment if required (grade D evidence).

### Concluding remarks

Beta-cell monogenic diabetes has been considered the strongest example of a precision medicine approach to diabetes treatment. However, this systematic review demonstrates that there is limited trial evidence to support precision treatment practices and many recommendations rely on case series and case reports. Importantly the strongest available evidence supports the existing practice consensus guidelines^4^ regarding GCK-related hyperglycemia and HNF1A-diabetes.

Randomized trials with long-term follow-up offer the strongest evidence but the low numbers of cases of each individual subtype make these very difficult to perform. We urge the medical community to publish the follow-up of the complete case series in all subtypes of beta-cell monogenic diabetes to establish the response to therapy, fill the identified gaps in precision treatment, and explore the precision treatment approaches to prevent complications. Future studies of precision treatment in beta-cell monogenic diabetes must account for the perspectives of people with monogenic diabetes, including the acceptability of the different medications in terms of route, patient-facing costs, and potential adverse effects. Cost-effectiveness analyses of the newer diabetes medications also need to be carried out. Finally, there must be purposeful efforts toward equity in achieving optimal diabetes outcomes for individuals living with diabetes. This includes screening approaches and “high-throughput pipelines that can be disseminated in every country worldwide”^128^. There must be special attention to groups of people and countries underrepresented in studies of monogenic diabetes to inform on potential differences in phenotype, treatment response, and precision medicine approaches.

### Supplementary information

Supplementary information

Description of Additional Supplementary Files

Supplementary Data 1

Supplementary Data 2

Reporting Summary

## Data Availability

All data used in this systematic review are publically available in the published scientific literature. Search strategies used for each monogenic diabetes subtype are detailed in Supplemental Tables 1–5. PRISMA figures were generated in Covidence. Excel files of the included studies corresponding to each PRISMA diagram are available as supplemental data (Supplementary Data 2).
