# Supplementary material for: Precision treatment of beta-cell monogenic diabetes: a systematic review
Source: Commun Med (Lond). 2024 Jul 18;4:145. doi: 10.1038/s43856-024-00556-1 (PMC11258280; doi:10.1038/s43856-024-00556-1)
Supplement: Supplementary file 1 — Supplementary information [file 43856_2024_556_MOESM1_ESM.pdf]

SUPPLEMENTAL ONLINE INFORMATION

Systematic Review of Treatment of Beta-Cell Monogenic Diabetes

Rochelle N. Naylor, Kashyap A. Patel, Jarno L.T. Kettunen, Jonna M.E. Männistö, Julie Støy, Jacques Beltrand, Michel Polak, ADA/EASD PMDI, Tina Vilsbøll, Siri A.W. Greeley, Andrew T. Hattersley, Tiinamaija Tuomi

Table of contents	Page
Supplemental Table 1. Search term for studies of GCK-related hyperglycemia, HNF1A-diabetes, and HNF4A-diabetes	2
Supplemental Table 2. Search term for studies of HNF1B-diabetes	3
Supplemental Table 3. Search term for studies of mitochondrial diabetes	4
Supplemental Table 4. Search term for studies of 6q24-diabetes	5
Supplemental Table 5. Search term for studies of SLC19A2-diabetes	6
Supplemental Table 6. Summary of included case report studies for GCK-related Hyperglycemia	7
Supplemental Table 7. Summary of included case report studies for HNF1A-diabetes and HNF4A-diabetes	8
Supplemental Table 8. Summary of included case report studies for 6q24-diabetes with use of non-insulin therapies during neonatal phase	9
Supplemental Table 9. Summary of included case report studies for 6q24-diabetes with use of insulin therapies during relapse phase	10
Supplementary Table 10. Summarized case report data on response to thiamine for SLC19A2-diabetes (TRMA syndrome)	11
Supplemental Figure 1	12
Supplemental Figure 2	13
Supplemental Figure 3	14
Supplemental Figure 4	15

Supplemental Table 1: Search terms and keywords used to identify relevant studies for GCK-related hyperglycemia, HNF1A-diabetes and HNF4A-diabetes

**Diabetes Phenotypes**	Monogenic diabetes; MODY; Maturity onset diabetes of the young
**Gene Names**	Hepatocyte Nuclear Factor 1 Alpha; HNF1; Hepatocyte Nuclear Factor 1-Alpha; Transcription Factor HNF-1; Hepatic Nuclear Factor 1 Alpha; HNF1alpha; HNF-1-Alpha; HNF1alpha; HNF1a; MODY3; HNF1A; TCF-1; TCF1; MODY-3; Hepatocyte Nuclear Factor 4 Alpha; HNF4; Hepatocyte Nuclear Factor 4-Alpha; Transcription Factor HNF-4; Hepatic Nuclear Factor 4 Alpha; HNF4alpha; HNF-4-Alpha; HNF4alpha; HNF4a; MODY1; HNF4A; TCF-14; TCF14; MODY-1; Glucokinase; HK4; Glucokinase (Hexokinase 4); Hexokinase Type IV; Hexokinase-4; HK IV; Hexokinase D, Pancreatic Isozyme; Hexokinase 4; Hexokinase-D; MODY2; GCK; MODY-2
**Treatments**	Metformin; Biguanide; Sulphonylurea; Sulfonylurea; Gliclazide; Glipizide; Glibenclamide; Glyburide; Glimepiride; Tolbutamide; Chlorpropamide; Repaglinide; Nateglinide; Thiazolidinedione; PPARg Agonist; Rosiglitazone; Pioglitazone; Troglitazone; Alphaglucosidase inhibitor; Acarbose; DPP4 inhibitor; DPP-4 inhibitor; Dipeptidylpeptidase 4 inhibitor; Dipeptidylpeptidase-4 inhibitor; Sitagliptin; Vildagliptin; Saxagliptin; Linagliptin; Alogliptin; SGLT2 inhibitor; SGLT2i; SGLT-2 inhibitor; SGLT-2i; Sodium Glucose Transporter 2 inhibitor; Dapagliflozin; Empagliflozin; Ertagliflozin; Canagliflozin; GLP1RA; GLP1 Receptor Agonists; GLP-1 Receptor Agonists; GLP-1RA; Exenatide; Liraglutide; Lixisenatide; Semaglutide; Dulaglutide; Albiglutide; Isophane; NPH insulin; Basal Insulin; Long acting insulin; Glargine; Detemir; Degludec; Insulin; Lifestyle; Diet; Dietary; Weight Loss; Exercise; Bariatric surgery; Obesity Surgery; Weight reduction surgery; gastric band; Roux-en-Y; Gastric sleeve; Gastric bypass
**Outcomes**	HbA1c; Treatment response; Glycemia; Treatment failure; Intolerance; Adverse effect; Side effect; Diarrhea; Lactic acidosis; Acute kidney injury; Hypoglycemia; Weight gain; Edema; Ketosis; Ketoacidosis; Pancreatitis; Nausea; Vomiting; Cardiovascular disease; Myocardial infarction; Revascularisation; Acute coronary syndrome; Coronary heart disease; Heart failure; Cardiac Failure; Angioplasty; Percutaneous Coronary Intervention; Cardiovascular Mortality; MACE; Stroke; TIA; Nephropathy; Proteinuria; Macroalbuminuria; Renal impairment; Chronic kidney disease; Microalbuminuria; Retinopathy; Neuropathy

Overall Search in PubMed, MEDLINE and Embase: (diabetes phenotype AND each gene AND treatment AND outcome) using US and UK spellings, from 1992 and English language only.

Supplemental Table 2: Search terms and keywords used to identify relevant studies for HNF1B-diabetes

**Diabetes Phenotypes**	MODY; monogenic diabetes; MODY5; MODY-5; HNF1B-MODY; HNF1B-diabetes; HNF1B diabetes; HNF1B disease; HNF1B-disease; HNF1B-related disease; renal cysts and diabetes; RCAD
**Gene Names**	HNF1 Homeobox B; vHNF1; Hepatocyte Nuclear Factor 1-Beta; Hepatocyte Nuclear Factor 1β; Hepatocyte Nuclear Factor 1-β; HNF-1-Beta; HNF1-β; HNF-1β; HNF1beta; HNF-1B; MODY5; TCF-2; TCF2; HNF1 Beta; HNF1B; MODY-5; hepatocyte nuclear factor 1b; hepatocyte nuclear factor 1 beta; hepatocyte nuclear factor 1 β
**Treatments**	Metformin; Biguanide; Sulphonylurea; Sulfonylurea; Gliclazide; Glipizide; Glibenclamide; Glyburide; Glimepiride; Tolbutamide; Chlorpropamide; Repaglinide; Nateglinide; Thiazolidinedione; PPARg Agonist; Rosiglitazone; Pioglitazone; Troglitazone; Alphaglucosidase inhibitor; Acarbose; DPP4 inhibitor; DPP-4 inhibitor; Dipeptidylpeptidase 4 inhibitor; Dipeptidylpeptidase-4 inhibitor; Sitagliptin; Vildagliptin; Saxagliptin; Linagliptin; Alogliptin; SGLT2 inhibitor; SGLT2i; SGLT-2i; Sodium Glucose Transporter 2 inhibitor; Dapagliflozin; Empagliflozin; Ertagliflozin; Canagliflozin; GLP1RA; GLP1 Receptor Agonists; GLP-1 Receptor Agonists; GLP-1RA; Exenatide; Liraglutide; Lixisenatide; Semaglutide; Dulaglutide; Albiglutide; Isophane; NPH insulin; Basal Insulin; Long acting insulin; Glargine; Detemir; Degludec; Insulin; Lifestyle; Diet; Dietary; Weight Loss; Exercise; Bariatric surgery; Obesity Surgery; Weight reduction surgery; Roux-en-Y; Gastric sleeve; Gastric bypass

Overall Search in PubMed, MEDLINE and Embase: (diabetes phenotype AND each gene AND treatment) using US and UK spellings, from 1992 and English language only

Supplemental Table 3: Search terms and keywords used to identify relevant studies for mitochondrial diabetes

**Diabetes Phenotypes**	MIDD*; MELAS and (diabetes); MELAS AND diabetes OR hyperglycemia OR hyperglycaemia OR dysglycemia OR dysglycaemia; mitochondrial diabetes; maternally inherited diabetes; maternally inherited diabetes and deafness; maternal diabetes and deafness; Mitochondrial encephalopathy, lactic acidosis, and stroke-like episodes AND diabetes OR hyperglycemia OR hyperglycaemia OR dysglycemia OR dysglycaemia; Mitochondrial encephalopathy, lactic acidosis, and stroke like episodes AND diabetes OR hyperglycemia OR hyperglycaemia OR dysglycemia OR dysglycaemia
**Gene Names**	MTTL1; Mitochondrially Encoded TRNA-Leu; Mitochondrially Encoded TRNA Leucine 1; MT-TL1; MTTE; MT-TE; Mitochondrially Encoded TRNA-Glu; Mitochondrially Encoded TRNA Glutamic Acid; MTTK; Mitochondrially Encoded TRNA-Lys; Mitochondrially Encoded TRNA Lysine; MT-TK; m.3243A; mt.3243; A3243G; m.3243A>G; mt.3243A>G;3243A-G; 3243 a to g; m. 14709; mt. 14709; T14709C; m. 14709T>C; mt. 14709T; 14709T-C; 14709 t to c; m.8396; mt.8396; A8396G; m.8396A; mt.8396A>G; 8396A-G; 8396 a to g; 3243; 14709; 8396;
**Diabetes Treatments**	Metformin; Biguanide; Sulphonylurea; Sulfonylurea; Gliclazide; Glipizide; Glibenclamide; Glyburide; Glimepiride; Tolbutamide; Chlorpropamide; Repaglinide; Nateglinide; Thiazolidinedione; PPARg Agonist; Rosiglitazone; Pioglitazone; Troglitazone; Alphaglucosidase inhibitor; Acarbose; DPP4 inhibitor; DPP-4 inhibitor; Dipeptidylpeptidase 4 inhibitor; Dipeptidylpeptidase-4 inhibitor; Sitagliptin; Vildagliptin; Saxagliptin; Linagliptin; Alogliptin; SGLT2 inhibitor; SGLT2i; SGLT-2i; Sodium Glucose Transporter 2 inhibitor; Dapagliflozin; Empagliflozin; Ertagliflozin; Canagliflozin; GLP1RA; GLP1 Receptor Agonists; GLP-1 Receptor Agonists; GLP-1RA; Exenatide; Liraglutide; Lixisenatide; Semaglutide; Dulaglutide; Albiglutide; Isophane; NPH insulin; Basal Insulin; Long acting insulin; Glargine; Detemir; Degludec; Insulin; Lifestyle; Diet; Dietary; Weight Loss; Exercise; Bariatric surgery; Obesity Surgery; Weight reduction surgery; gastric band; Roux-en-Y; Gastric sleeve; Gastric bypass;
**Other Treatments 1**	statin(s); fibrate(s); PCSK9-inhibitor(s); ezetimibe; simvastatin; rosuvastatin; atorvastatin; pravastatin; Fluvastatin
**Other Treatments 2**	Vitamin(s); dietary supplements; ubiquinol; ubiquinone; ubidecarenone; coenzyme Q; CoQ_{10}

*Not monoclonal Ig deposition disease, model informed drug development

Overall Search in PubMed, MEDLINE and Embase: (diabetes phenotype and genetic variant AND (diabetes treatment OR Other Treatments 1 OR Other Treatments 2) using US and UK spellings, from 1992 and English language only

Supplemental Table 4: Search terms and keywords used to identify relevant studies for 6q24 transient neonatal diabetes

**Diabetes Phenotypes**	Diabetes Mellitus, Permanent Neonatal; Diabetes Mellitus, Transient Neonatal, 3; 6q24-Related Transient Neonatal Diabetes Mellitus; Diabetes Mellitus, Neonatal, with Congenital Hypothyroidism; Diabetes Mellitus, Permanent Neonatal, with Cerebellar Agenesis; Diabetes Mellitus, Transient Neonatal, 2; Diabetes Mellitus, Transient Neonatal, 1; maturity-onset diabetes of the young; MODY; diabetes mellitus/genetics; monogenic diabetes; neonatal diabetes; KCNJ11-diabetes; ABCC8-diabetes; KCNJ11-PNDM; ABCC8-PNDM; infancy-onset diabetes; NDM; PNDM; TNDM; 6q24-related diabetes mellitus; 6q24-related diabetes; maturity onset diabetes; maturity-onset diabetes; MODY; 6q24-TNDM; 6q24 TNDM; iTND; DMTN;
**Gene Names**	PLAGL1 protein, human; HYMAI, RNA; PLAGL1; HYMAI; ZPF57; 6q24; chromosome 6q24; UPD6; Uniparental Disomy of Chromosome 6

Overall Search in PubMed, MEDLINE and Embase: (diabetes phenotype AND each gene) from 1992 in humans and English language only

Supplemental Table 5: Search terms and keywords used to identify relevant studies for SLC19A2-diabetes

**Diabetes Phenotypes**	Thiamine responsive megaloblastic anemia syndrome; megaloblastic anaemia and deafness; thiamine-responsive anemia; Thiamine-responsive megaloblastic anemia; thiamine-responsive anemia; Rogers syndrome; TRMA; Thiamine-responsive megaloblastic anaemia syndrome; diabetes mellitus and sensorineural deafness and megaloblastic anemia; diabetes mellitus and sensorineural deafness; megaloblastic anaemia; Thiamine-responsive megaloblastic anemia syndrome with the addition: thiamine-responsiv*, thiamine responsiv*, thiamineresponsiv*, roger's syndrome*, roger's disease*, thiamine-dependent, thiaminedependent, rogers syndrome*, rogers disease*, abboud disease*, abboud syndrome*, thiamine transport and metabolism
**Gene Names**	SLC19A2; solute carrier family 19 (thiamine transporter), Member 2; thiamine transporter 1; ThTr-1; THTR1; THT1; THT-1; TRMA; TC1; TC-1; reduced folate carrier protein (RFC) like; high affinity thiamine transporter; thiamine carrier 1; THMD1; THMD-1; ThTr1; THTR-1; thiamine transport; solute carrier family 19 (thiamine transporter), Member 2; SLC19A2 protein, human

*Denotes use of wildcard character to search for all terms that begin with that word

Overall Search in PubMed, MEDLINE and Embase: (diabetes phenotype AND each gene) from 1992 in humans and English language only

Supplemental Table 6. Summary of included case reports for GCK-related hyperglycemia

GCK-related hyperglycemia Case reports (or single-subject data extracted from studies)							
Study ID	Sex	Age (years) at Diabetes Diagnosis/Assessment	Baseline treatment	Comparison	Pre-HbA1c (%)	Post-HbA1c (%)	Interval between pre/post HbA1c (months)
Almeida 2014	F	9/12	No pharmacologic therapy	No pharmacologic therapy	6.3	5.9	36
Carmody 2015	M	4/15	Insulin	No pharmacologic therapy	6.0-6.8	6.2-6.8	11
DellaManna 2012	M	11/21	No pharmacologic therapy	No pharmacologic therapy	6.3	5.9	102
	F	1/10.5	No pharmacologic therapy	No pharmacologic therapy	6.6	6.9	126
Ebrahim 2014	F	14/Unk*	No pharmacologic therapy	SU (given for exercise-induced hyperglycemia)	6.7	5.8	24
Loomba-Albrecht 2010	M	3/15	Insulin	SU	6.7	6.5	9
Papadimitriou 2015	M	5/12	No pharmacologic therapy	No pharmacologic therapy	6.5	7.1	84
Talapatra 2008	F	25/Unk*	Insulin	No pharmacologic therapy	6.2	6.0	12

*Unknown. OHA, oral hypoglycemia agents; SU, sulfonylurea; DPP4i, DPP4inhibitor; GLP1RA, GLP1 receptor agonist; TZD, thiazolidinedione

Supplemental Table 7. Summary of included case reports for HNF1A-diabetes and HNF4A-diabetes

HNF1A-diabetes Case reports (or single-subject data extracted from studies)							
Study ID	Sex	Age (years) at Diabetes Diagnosis/Assessment	Baseline treatment	Comparison	Pre-HbA1c (%)	Post-HbA1c (%)	Interval between pre/post HbA1c (months)
Ahluwalia 2009	M	14/39	SU+Metformin	GLP-1RA	7.7	6.2	10
Becker 2014	F	13/14	None	Glinides	7.4	5.6	6
	M	14/14	SU	Glinides	8.5	6.2	6
	F	11/11	Insulin	Glinides + insulin	8.6	8.2	6
Dashora 2012	F	22/57	SU + metformin	SU + DPP4i	9.5	7.4	15
Fang 2015	F	19/19	Insulin	SU	7.6	6.5	3
Globa 2017	NR*	12/13	Metformin	SU + DPP4i	7.6	6.3	3
	NR	12/16	Insulin+DPP4i+metformin	SU	8.4	6.8	3
	NR	13/14	Insulin+DPP4i+metformin	SU + DPP4i	8.2	6.7	3
	NR	16/17	Insulin	SU	6.8	5.8	3
	NR	15/16	Metformin	SU	8.4	6.1	3
	NR	14/17	Metformin+DPP4i	SU	8.9	6.6	3
Habeb 2011	M	7/7	None	SU	7.2	6.5	21
Ješić 2008	F	10/10	None	SU	7.9	5.8	3
Katra 2010	F	32/39	SU+metformin	SU+metformin+DPP4i	7.2	6.3	3
	F	21/62	SU+insulin	SU+insulin+DPP4i	8.8	6.3	3
Khelifa 2016	F	14/26	Insulin	SU	10.8	7.3	6
Lumb 2009	F	18/57	SU + TZD	SU + TZD + DPP4i*	9.6	8.7	12
Oliveira 2021	F	13/19	None	SU	6.8	5.5	24
Pearson 2000	M	20/33	Metformin	SU restarted**	10.3	5.3	6
	M	21/26	Metformin	SU**	7.9	4.8	6
Shepherd 2009	F	15/33	Insulin	SU	7.4	7.4	3
Urakami 2015	F	12/12	Insulin	GLP-1RA + SU ***	8.9	7.1	3
			GLP-1RA + SU	GLP-1RA	7.1	Range: 6.8-7.5<br>Last value: 7.5	33
HNF4A-diabetes Case reports (or single-subject data extracted from studies)							
Globa 2017	NR	13/15	None	SU	8.8	7.5	3
	NR	17/17	Metformin	SU	7.2	6.0	3

*Not reported. **TZD was changed from rosiglitazone to pioglitazone at 3 months with HbA1c increase from 7.7% to 8.7%. ***In both cases individuals were taken off SU and had deterioration of HbA1c, which improved when SU was restarted;****After molecular diagnosis, insulin weaned off when liraglutide started. SU added while GLP1-RA was weaned up, and then SU was discontinued.

Supplemental Table 8. Summary of included case report studies for 6q24-diabetes with use of non-insulin therapies during neonatal phase

6q24-diabetes Case reports with use of non-insulin therapies during neonatal phase (n=16)								
Study ID	6q24 Mechanism (UPD6, PD, MD, NR)	Sex	Birth weight (grams)	Age at Diagnosis of DM (days)	SU successful Yes/No	Days until insulin stopped	Maximum SU dose (G, mg/kg/d)	Age at remission of diabetes (weeks)
Gore 2020	PD	F	1820	1	Yes	5	1	7
Garcin 2018	UPD6	M	1370	1	Yes	3	0.15	18
	PD	F	NR	1	No	NA	4	NR
Li 2018	UPD6	NR	NR	25	Yes	NR	0.52	16
	UPD6	NR	NR	4	Yes	13	0.4	12
	PD	NR	NR	8	No	NA	NR	30
	UPD6	NR	NR	2	No	NA	NR	NA*
Neumann 2018	UPD6	M	1700	1	Yes	26	1.2	16
Cao 2017	UPD6	F	2000	1	No	NA	NR	NA*
Senguttuvan 2015	PD	M	NR	2	No	NA	NR	12
Zhang 2015	MD	F	1740	24	Yes	5	0.09	NR
Carmody 2014	NR	NR	NR	14	Yes	14	NR	6
	NR	NR	NR	4	Yes	11	NR	4
	NR	NR	NR	1	Yes	1	NR	14
Yao 2014	UPD6	F	2000	2	No	7	0.1	NA*
Hewes 2010	NR	M	2720	8	No	NA	NR	12

NR, Not reported; NA, Not applicable; G, Glibenclamide; UPD6, Uniparental paternal disomy of chromosome 6; PD, Paternally-inherited duplication involving 6q24; MD, Maternal methylation defect (either ZFP57 mutations or unknown cause); NA*, continued to require insulin as old as 41-60 months.

Supplemental Table 9. Summary of included case report studies for 6q24-diabetes with use of non-insulin therapies during relapse phase

6q24-diabetes Case reports with use of non-insulin therapies during relapse phase (n=14)												
Study ID	6q24 Mechanism (UPD6, PD, MD, NR)	Sex	Birth weight (grams)	Age at diagnosis of DM (days)	Age at remission of neonatal DM (weeks)	Age at DM relapse (years)	Insulin dose prior to NIT (U/kg/d)	Age at start of NIT (years)	NIT Used	Insulin discontinued Yes/No	HbA1c before NIT	HbA1c after NIT
Kontbay 2022	MD	F	3000	1	12	8.5	0.25	8.5	Glib	Yes	NR	8.7
Sato 2021	PD	M	2226	2	26	14.5	0	16	Glib	Yes**	6.7	NR
von dem Berge 2021	MD	NR	NR	8	6	14	0.7	14	Glib	Yes	8.2	5.7
Uchida 2020	PD	F	1765	11	8	10.8	0	10.8	Met	Yes	7.4	6.5
Fu 2019	MD	M	1700	45	12	14	0.2	14	Glim	Yes	7.8	6.5–7
Garcin 2018	UPD6	F	NR	1	32	12	0	12	Glib	Yes**	NR	NR
Carmody 2015	UPD6	F	1280	1	16	13	0.6	20	Glib, Sit, Met	Yes	8.2	7.1
	UPD6	M	2470	1	28	12	0.7	23	Glib, Sit, Met	Yes	7.8	6.6
	UPD6	F	2240	1	24	27	0.4	29	Glib	Yes	7.2	7.3
	UPD6	F	1810	1	12	12	0.8	28	Glib	Yes	9.9	7.5
Yorifuji 2014	PD	M	1660	7	28	12	0	12	Vog, Alo	Yes**	7.5	6.2
Sovik 2012	PD	M	1840	6	12	Adult	0	43	Met, Glib	Yes**	NR	9.0
	PD	F	1440	12	54	11	0.5	28	Met	No	8.8	10.0
Schimmel 2009	UPD6	M	2060	2	28	15	0	15	Glim	Yes**	NR	5.7–7.8

NR, Not reported; NA, Not applicable; UPD6, Uniparental paternal disomy of chromosome 6; PD, Paternally-inherited duplication involving 6q24; MD, Maternal methylation defect (either ZFP57 mutations or unknown cause); NIT, Non-insulin therapy; Glib, Glibenclamide (glyburide); Glim, Glimepiride; Vog, Voglibose (alpha-glucosidase inhibitor); Alo, Alogliptin (DPP4-inhibitor); Met, Metformin; Sit, Sitagliptin; Yes**, insulin never given during relapse phase.

Supplemental Table 10. Summarized data on response to thiamine from case report studies for SLC19A2-diabetes (TRMA syndrome)

SLC19A2-diabetes Case reports combined (n=44)	Sex (M/F)	Median age at DM diagnosis (range) (y)	Median dose (range) at DM diagnosis (mg/d)	Median (range) duration of Thiamine at latest follow-up	Median HbA1c levels (%) (range)		Outcome, specified	Response to Thiamine Yes, % (n)
		Median age at Thiamine start (range) (y)	Median max dose (range) (mg/d)		Pre	Post	Proportions (%) in each group of all	
	15/29	1.15 (0.2–5.4)	100 (25–200)	0.9 (2 days–25 years)	8.4 (5.0–21.0)	6.6 (4.5–10)	25% Insulin-independent, n=11<br>34% Reduced insulin dose, n=15<br>14% Insulin not started, n=6<br>7% Improved glycemic control, n=3<br>20% No response, n=9	80 (35)
		2.4 (0.2–19)	100 (25–600)					

Supplemental Figure 1. GCK-related hyperglycemia, HNF1A-diabetes and HNF4A- diabetes

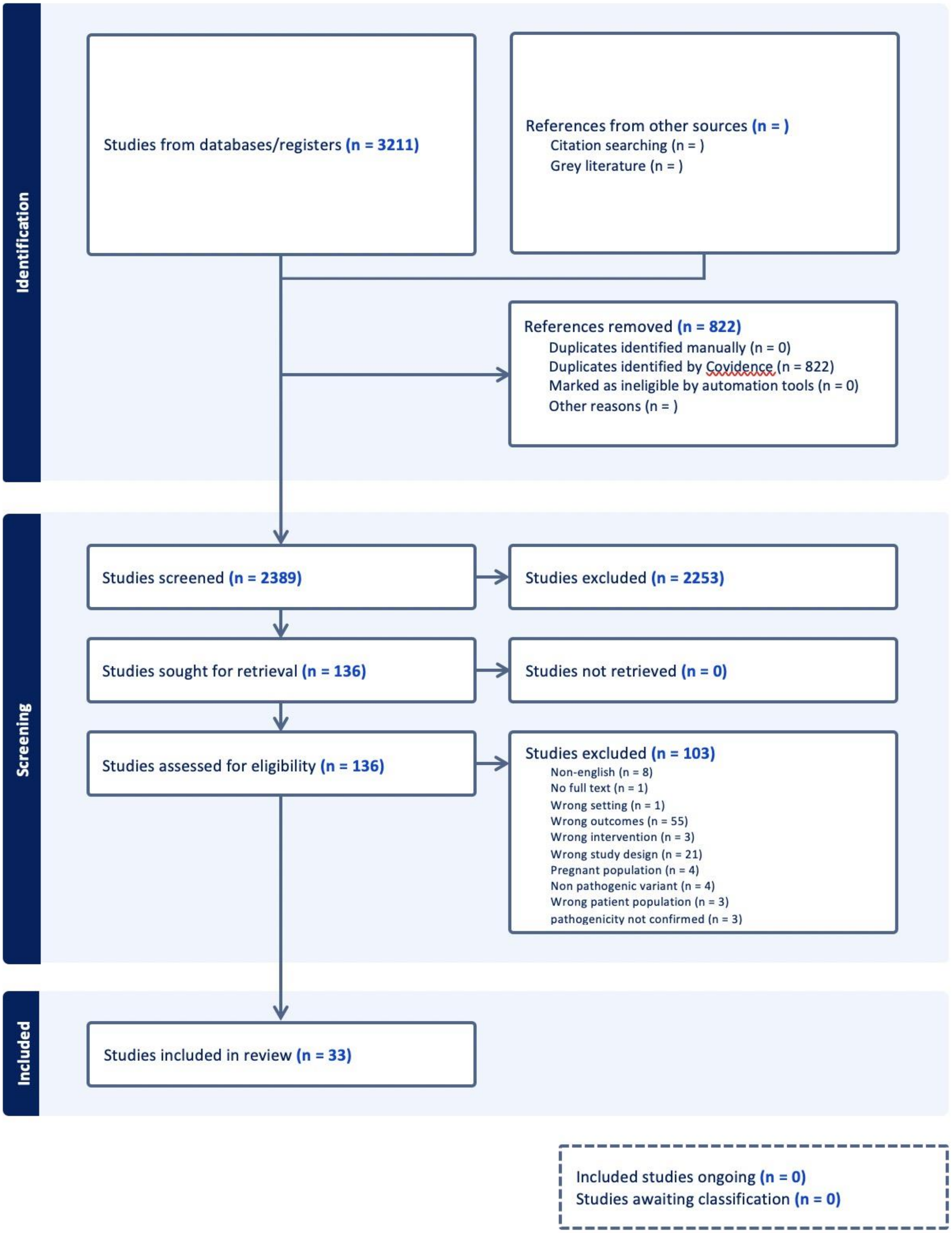

PRISMA search summary for GCK-related hyperglycemia, HNF1A-related diabetes and HNF4A-related diabetes. Search dates: PubMed June 28, 2021, Embase June 9, 2021

Supplemental Figure 2. HNF1B-diabetes and mitochondrial diabetes

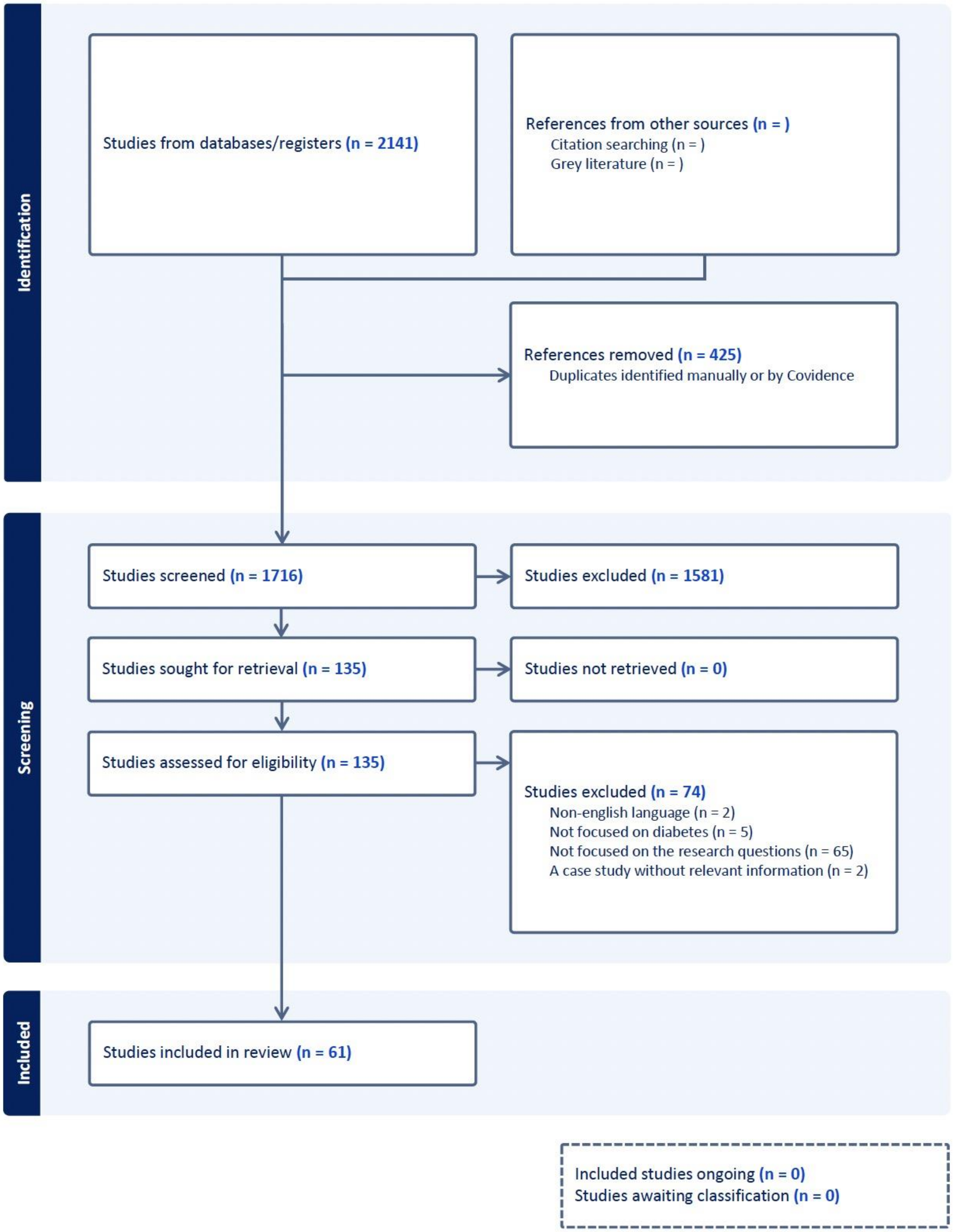

PRISMA search summary for HNF1B-MODY and mitochondrial diabetes. Search dates: HNF1B-MODY, PubMed June 23, 2021, Embase June 17, 2021; MD, PubMed February 9, 2022, Embase February 14, 2022

Supplemental Figure 3. 6q24-related transient neonatal diabetes

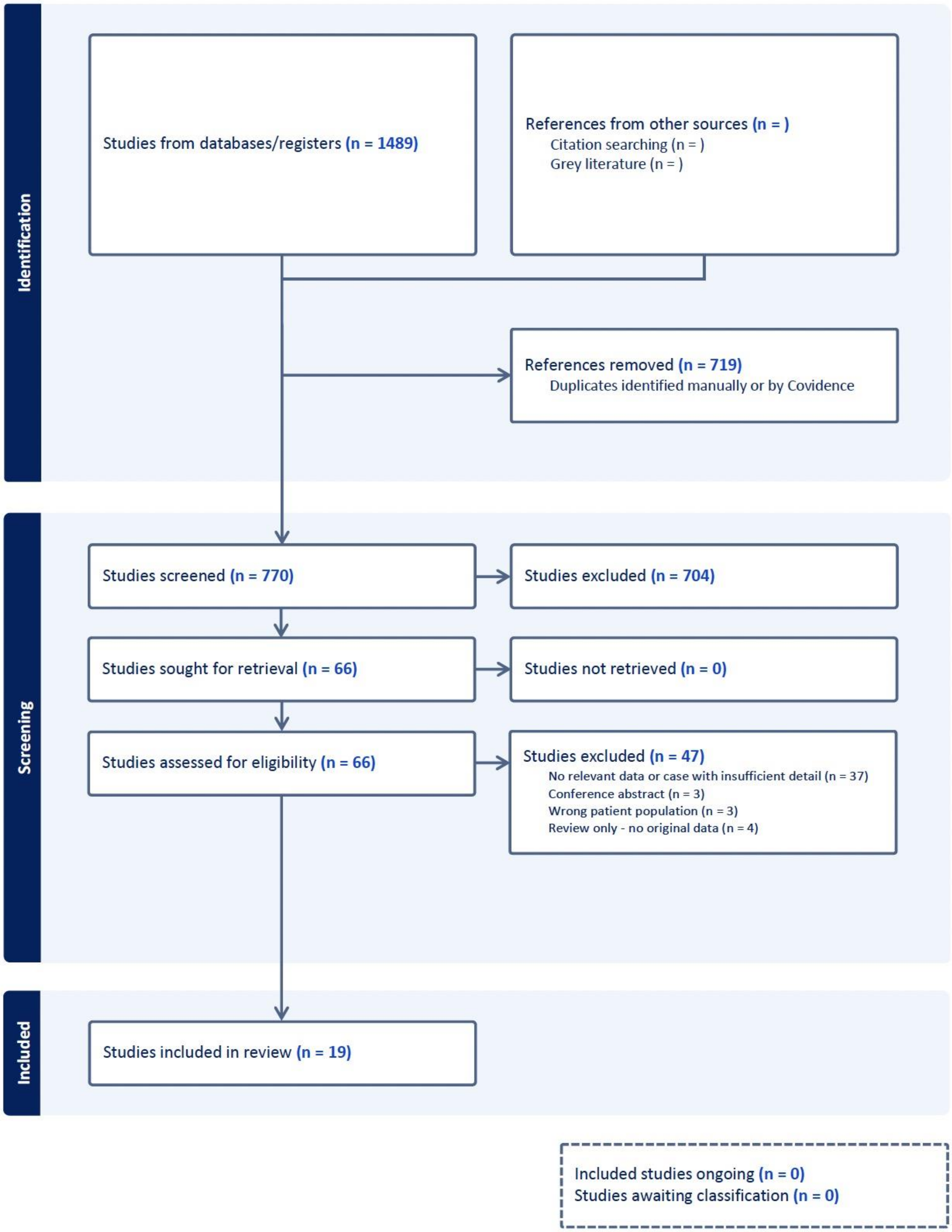

PRISMA search summary for 6q24-related transient neonatal diabetes. Search dates: PubMed Sept 24, 2022, Embase Sept 20, 2022.

Supplemental Figure 4. SLC19A2-diabetes

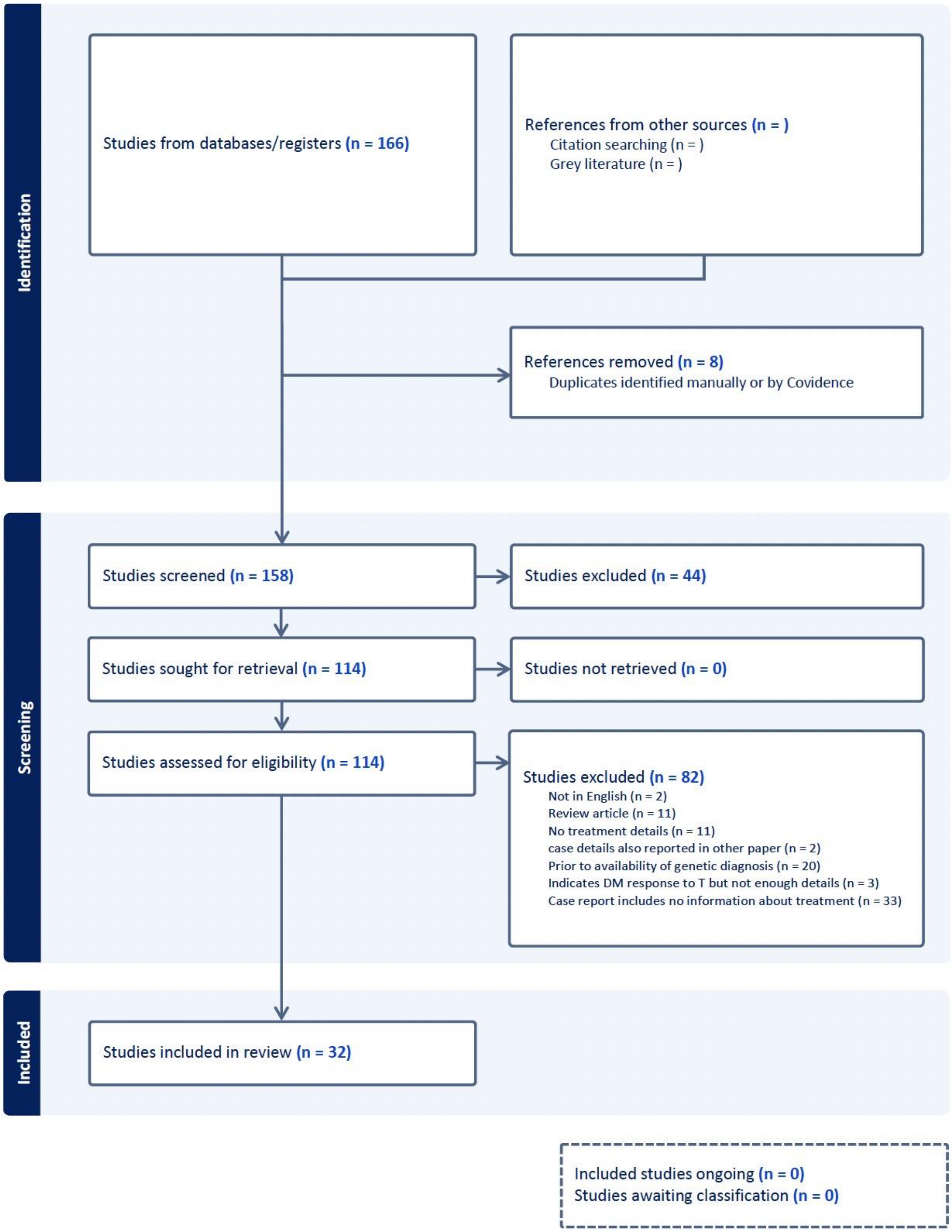

PRISMA search summary for SLC19A2-related diabetes. Search dates: PubMed Feb 9, 2022, Embase Feb 14, 2022
