# Supplementary material for: Precision treatment of beta-cell monogenic diabetes: a systematic review
Source: Commun Med (Lond). 2024 Jul 18;4:145. doi: 10.1038/s43856-024-00556-1 (PMC11258280; doi:10.1038/s43856-024-00556-1)
Supplement: Supplementary file 2 — Description of Additional Supplementary Files [file 43856_2024_556_MOESM2_ESM.pdf]

Description of Additional Supplementary Files

File name: Supplementary Data 1

File Description: SLC19A2-diabetes case report

File name: Supplementary Data 2

File Description: PRISMA diagram are available as supplemental data
